# Characterization of Greenbeard Genes Involved in Long-Distance Kind Discrimination in a Microbial Eukaryote

**DOI:** 10.1371/journal.pbio.1002431

**Published:** 2016-04-14

**Authors:** Jens Heller, Jiuhai Zhao, Gabriel Rosenfield, David J. Kowbel, Pierre Gladieux, N. Louise Glass

**Affiliations:** 1 The Plant and Microbial Biology Department, The University of California, Berkeley, California, United States of America; 2 INRA, UMR BGPI, F-34398 Montpellier, France; Duke University Medical Center, UNITED STATES

## Abstract

Microorganisms are capable of communication and cooperation to perform social activities. Cooperation can be enforced using kind discrimination mechanisms in which individuals preferentially help or punish others, depending on genetic relatedness only at certain loci. In the filamentous fungus *Neurospora crassa*, genetically identical asexual spores (germlings) communicate and fuse in a highly regulated process, which is associated with fitness benefits during colony establishment. Recognition and chemotropic interactions between isogenic germlings requires oscillation of the mitogen-activated protein kinase (MAPK) signal transduction protein complex (NRC-1, MEK-2, MAK-2, and the scaffold protein HAM-5) to specialized cell fusion structures termed conidial anastomosis tubes. Using a population of 110 wild *N*. *crassa* isolates, we investigated germling fusion between genetically unrelated individuals and discovered that chemotropic interactions are regulated by kind discrimination. Distinct communication groups were identified, in which germlings within one communication group interacted at high frequency, while germlings from different communication groups avoided each other. Bulk segregant analysis followed by whole genome resequencing identified three linked genes (*doc-1*, *doc-2*, and *doc-3*), which were associated with communication group phenotype. Alleles at *doc-1*, *doc-2*, and *doc-3* fell into five haplotypes that showed transspecies polymorphism. Swapping *doc-1* and *doc-2* alleles from different communication group strains was necessary and sufficient to confer communication group affiliation. During chemotropic interactions, DOC-1 oscillated with MAK-2 to the tips of conidial anastomosis tubes, while DOC-2 was statically localized to the plasma membrane. Our data indicate that *doc-1*, *doc-2*, and *doc-3* function as “greenbeard” genes, involved in mediating long-distance kind recognition that involves actively searching for one’s own type, resulting in cooperation between non-genealogical relatives. Our findings serve as a basis for investigations into the mechanisms associated with attraction, fusion, and kind recognition in other eukaryotic species.

## Abstract 2

This study identifies variable "greenbeard" genes in wild populations of the filamentous fungus *Neurospora crassa* that affect recognition and chemotropic interactions between germlings and thereby separate the fungal population into communication groups.

## Author Summary

Microorganisms undergo social activities that benefit the species, but for social microbes, the ability to discriminate between genetically similar and genetically dissimilar individuals is instrumental in preventing cheaters from taking advantage of altruistic behavior. While kin recognition is important in animals, microbes often use kind recognition, in which cells are genetically related only at certain loci—so-called “greenbeard” genes. Genomic and genetic analyses of a wild population of the filamentous fungus *Neurospora crassa* showed that greenbeard genes mediate long-distance kind discrimination that regulates communication and chemotropic interactions of cells prior to somatic cell fusion; *N*. *crassa* cells actively search for fusion partners with similar greenbeard genes. Kind discrimination was regulated by a set of highly divergent paralogous genes (*doc-1*, *doc-2*, and *doc-3*) that were necessary and sufficient to confer communication identity. Alleles that confer the interaction phenotype at the *doc* loci have been maintained through multiple speciation events, suggesting that selection is acting to maintain different communication groups in fungi.

## Introduction

Microbes engage in a wide variety of cooperative interactions to perform complex, multicellular, coordinated activities such as dispersal, foraging, nutrient acquisition (including virulence), organismal defense, and production of multicellular structures such as biofilms, networks, or fruiting bodies [1–4]. Unlike larger organisms, many of the processes involved in microbial cooperation take place extracellularly in the public space, so that public goods produced by cooperative cells are particularly vulnerable to exploitation by cheaters (which benefit from the availability of public goods without producing them) [3,5]. Microbes have evolved multiple mechanisms for enforcing cooperation, by performing differential actions to others (i.e., rewarding cooperators and/or penalizing cheaters) according to kinship (i.e., genome-wide relatedness) or kind (i.e., phenotypic similarity caused by genetic relatedness at certain loci) [1,6,7]. In fact, much discrimination in microbes appears to be based on kind rather than kin [3,7], and many of the frequency-dependent processes commonly observed in microbes can be interpreted as kind discrimination, as they depend on expressing a trait that has differential effects on bearers and non-bearers [8–11]. Under this model, cooperation can involve kin or non-kin individuals as long as they share a single cooperative gene or set of genes; such genes are termed “greenbeard” genes. Individuals with a given greenbeard gene can identify the presence of that greenbeard gene in other individuals, resulting in a change in activity or interaction [12].

Kind discrimination can be divided into “harming” and “helping” types [11]. “Harming” kind discrimination includes the poison—antidote system, which is widespread among bacteria and some archaea, and involves releasing a bacteriocin that can be rendered ineffective by related strains expressing an antidote protein, but which kills strains lacking it (reviewed in [13]). “Helping” kind discrimination is exemplified by the slime mold *Dictyostelium discoideum* or the yeast *Saccharomyces cerevisiae*, for which discrimination involves cell adhesion proteins that are important for adherence of amoeba in aggregation streams or for flocculation, respectively [7,14,15].

In many microbial eukaryotes, somatic growth is a form of cooperation: all somatic cells are “hopeful reproductives" (i.e., they retain the potential to sexually reproduce), but most of them will never engage in sexual reproduction and instead help other cells to reproduce, which provides a direct benefit to the individual, and an indirect benefit to related individuals upon somatic fusion [16]. However, with somatic fusion, soma becomes a public good that is vulnerable to exploitation by cheaters [17–19]. In filamentous fungi, somatic fusion can occur within or between clonemates: an interconnected mycelia network can be formed via cell fusion between germinated asexual spores (germlings) [20–22] and/or between hyphae in a mature colony. The benefits of fungal somatic fusion have been associated with the sharing of cytoplasm, organelles (including nuclei), nutrients, and other resources to ensure rapid spatial expansion [23–26], intra-organismal communication, mitotic recombination (especially for highly clonal species [27]), redistribution of water and nutrients, and general homeostasis within the mycelium [16,25,28–32].

Somatic fusion can also occur in filamentous fungi via hyphal fusion between different colonies, potentially leading to the presence of genetically different nuclei in a common cytoplasm (heterokaryon). The cost of somatic fusion has been associated with the transmission of infectious cytoplasmic elements and mycoviruses, which are widespread among fungi [33–35]. Within a heterokaryon, allorecognition processes determine the fate of the fused cells: compatible genotypes lead to a heterokaryon indistinguishable from a homokaryotic colony, while heterokaryotic cells resulting from the fusion of incompatible genotypes are rapidly compartmentalized and undergo programmed cell death, termed vegetative (or heterokaryon) incompatibility [36–39]. Empirically, vegetative incompatibility in filamentous fungi has been shown to prevent somatic parasitism and reduce the risk of transmission of selfish nuclei and cytoplasmic elements [40–42].

**Fig 1:**
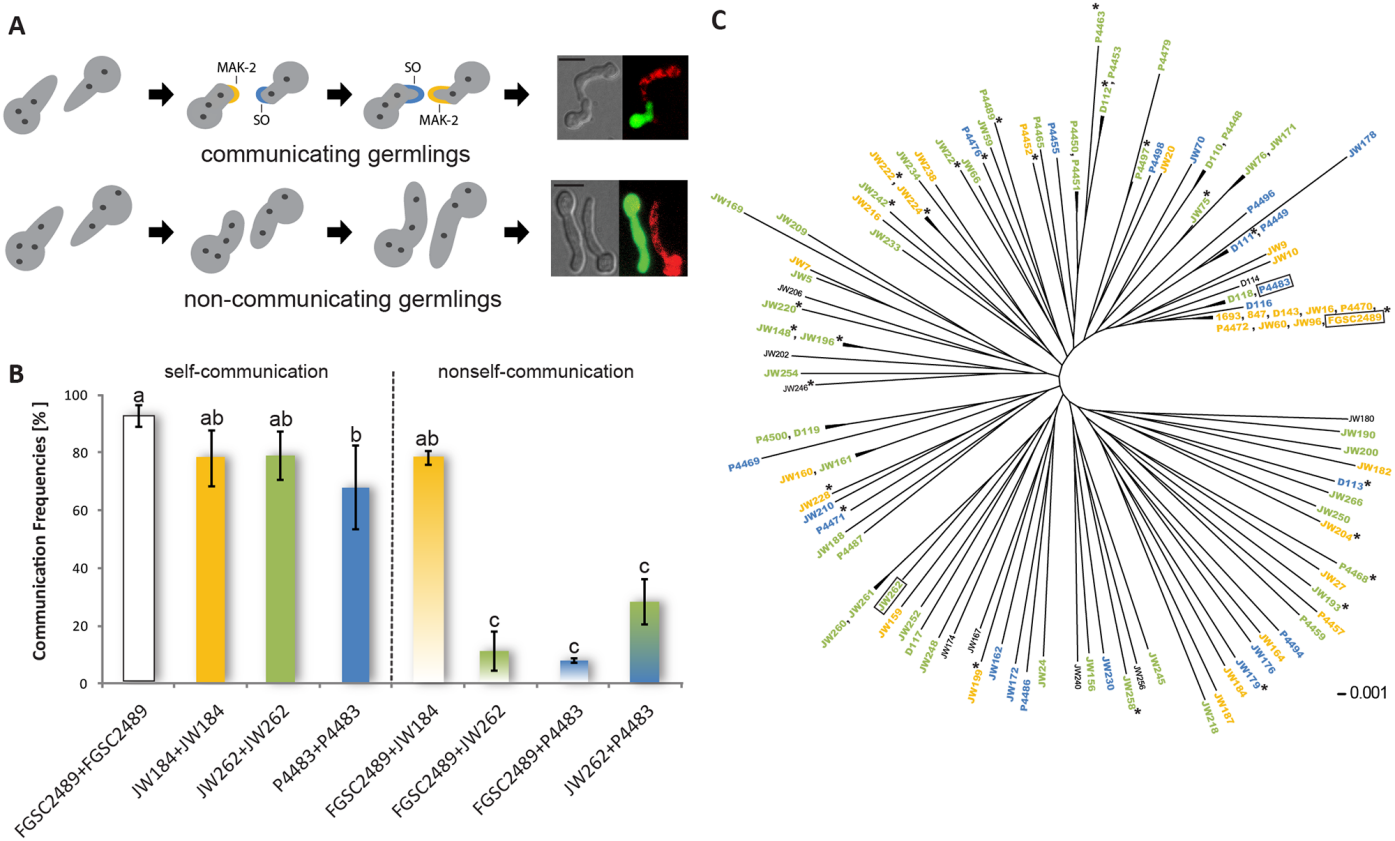
Communication groups (CGs) within a *Neurospora crassa* population from Louisiana. (A) Schematic visualization of communicating versus non-communicating germlings. Blue and yellow crescents show the out-of-phase oscillation pattern of the MAPK MAK-2 versus SOFT during chemotropic interactions between genetically identical cells (top panel); oscillation intervals (switch from MAK-2 accumulation at one tip to SOFT accumulation at the same tip) are 4–5 min [44]. Bottom panel shows interactions between cells of different communication group, in which oscillation and chemotropic interactions are significantly reduced. Micrographs on right show examples for communication (top) versus non-communication (bottom) in germlings expressing cytoplasmic green fluorescent protein (GFP) mixed with germlings stained with FM4-64 (Scale bars: 10 μm). (B) Self-communication versus non-self-communication frequencies between germlings from wild isolates. Communication phenotype was assessed 4 h after inoculation. One-color bars denote self-communication frequency between genetically identical germlings from a single strain, while two-color bars denote communication frequency between genetically different germlings. Experiments were performed in triplicate with at least 100 germling pairs counted for each experiment. Black bars indicate standard deviation. Different letters denote a statistically significant difference (one-way ANOVA with Scheffé multiple comparison calculation; *p* < 0.01; see S1 Data for numerical values). (C) Germlings of 110 *N*. *crassa* isolates were qualitatively tested for their ability to communicate with CG tester strains (FGSC 2489, CG1; JW262, CG2; P4483, CG3; framed). Isolates shown in orange communicated with the CG1 tester strain, isolates shown in green communicated with the CG2 tester strain, and isolates shown in blue communicated with the CG3 tester strain. Isolates shown in black did not produce enough spores or did not communicate well enough to analyze communication group affiliation. Note lack of population structure among the wild isolates. Tree modified from [52]. Asterisks indicate the 26 strains with genomic sequence available.

In *Neurospora crassa*, the molecular basis of chemotropic interactions and cell fusion between genetically identical germlings has been studied extensively, but processes involved in recognition between genetically non-identical germlings have not been investigated. In genetically identical germlings, chemotropic interactions are initiated when germlings are in close proximity (~15 μm), and are associated with redirected growth and cell fusion via specialized structures termed conidial anastomosis tubes (CATs) (Fig 1A) [21,43]. During chemotropic interactions, the mitogen-activated protein kinase (MAPK) signal transduction protein complex (NRC-1, MEK-2, MAK-2, and the scaffold protein HAM-5) assembles/disassembles at the CAT tip of communicating germlings with perfectly out of phase dynamics to SOFT, a scaffold protein for components of the MAPK cell wall integrity pathway [44–48]. For example, if SOFT is at the CAT tip of one germling, NRC-1/MEK-2/MAK-2/HAM-5 complex is at the CAT tip of its partner germling; switching between MAK-2 complex and SOFT at a single CAT tip occurs approximately every 4–5 min (Fig 1A). The spatiotemporal coordination of the MAK-2 signal transduction complex versus SOFT at CATs during chemotropic interactions is postulated to allow genetically and developmentally identical cells to coordinate their behavior and achieve mutual attraction and fusion, while avoiding self-stimulation [44,49].

Here, we investigate recognition interactions between genetically different germlings using a population of *N*. *crassa*. Within this population, we defined distinct communication groups and show that genetically different germlings can distinguish each other without physical contact, in a process that involves actively searching for one’s own type. Communication groups were associated with haplotypes at three linked loci, *doc-1*, *doc-2*, and *doc-3*. Alleles at *doc-1*, *doc-2*, and *doc-3* were highly divergent between haplotypes, and they showed transspecies polymorphisms consistent with long-term balancing selection caused by negative frequency-dependent selection (i.e., rare allele advantage). Live cell imaging showed that DOC-1 oscillates with the conserved MAK-2 signal transduction pathway. Thus, here we describe the identification and characterization of a form of assortative kind recognition that involves multiple alleles at the greenbeard genes *doc-1*, *doc-2*, and *doc-3* and that acts at a distance by preventing chemotropic interactions between non-kind germlings from different communication groups. Our findings reveal a heretofore underappreciated complexity in fungal communication and serve as a basis for investigations into mechanisms associated with long-distance kind recognition in other eukaryotic species.

## Results

### Identification of Communication Groups in *N*. *crassa* Populations

Kind discrimination was neglected in previous studies on germling communication in *N*. *crassa*, as strains were used whose genetic background was identical to the commonly used laboratory strain (FGSC 2489) [43,44,50]. To assess whether germlings of different genetic backgrounds can undergo productive chemotropic interactions, we took advantage of a *N*. *crassa* population isolated from Louisiana, United States; the laboratory strain (FGSC 2489) is a member of this population [51]. RNAseq data showed a substantial level of polymorphism (on order of two single nucleotide polymorphisms [SNPs] per kbp), while analyses of population structure revealed no subdivision [51–53]. We randomly picked 14 isolates from this population and analyzed chemotropic interactions (defined as reoriented growth of germlings toward each other; Fig 1A) between genetically identical germlings from the same isolate (self-communication) versus chemotropic interactions between germlings from the 14 isolates and FGSC 2489 (non-self-communication) using differential fluorescence labeling (see Materials and Methods). Self-communication frequencies among the wild isolates varied between approximately 50% and 95% (Figs 1A, 1B and S1). However, when germlings of these 14 wild isolates were paired with FGSC 2489 (non-self-communication), some pairings showed very low communication frequencies, while others showed communication frequencies with FGSC 2489 that were similar to self-communication frequencies (Figs 1A, 1B and S1). Importantly, this communication phenotype was not linked to the mating type of the strains (S1 Table). We therefore assessed self and non-self germling communication phenotype of the remaining members of the Louisiana population (95 strains) (see Materials and Methods; S1 Table). From these analyses, three communication groups were defined. While genetically identical and non-identical germlings within a communication group showed robust chemotropic interactions, germlings from different communication groups, even when in close proximity, grew past each other to find a germling of their own communication group (S1 and S2 Movies). The first communication group (CG1) contained 29 strains, which showed similar communication frequencies between and within strains and which included FGSC 2489 (Fig 1C, orange). The second communication group (CG2) contained 51 strains (Fig 1C, green), while the third communication group (CG3) contained 21 strains (Fig 1C, blue; S1 Table). The remaining nine strains (Fig 1C, black) did not produce sufficient asexual spores to determine CG affiliation. These observations indicated that the germling communication trait in *N*. *crassa* functioned in assortative kind recognition and occurs at a distance.

To determine whether communication groups are unique to the Louisiana population, we used tester strains for each communication group (FGSC 2489, CG1; JW262, CG2; P4483, CG3; framed in Fig 1C) and evaluated communication frequencies with other *N*. *crassa* population samples (isolates from Haiti, Panama, Costa Rica, Puerto Rico, Texas, Florida, Venezuela, Guyana). All of the wild isolates from these different *N*. *crassa* populations communicated with one of the three Louisiana communication group tester strains (CG1, CG2, or CG3; S1 Table). Thus, communication groups were not unique to the Louisiana population, but also occurred in other wild populations of *N*. *crassa*.

### Communication Group Affiliation Is Associated with Genomic Rearrangements and with Loci that Encode Highly Divergent Alleles

**Fig 2:**
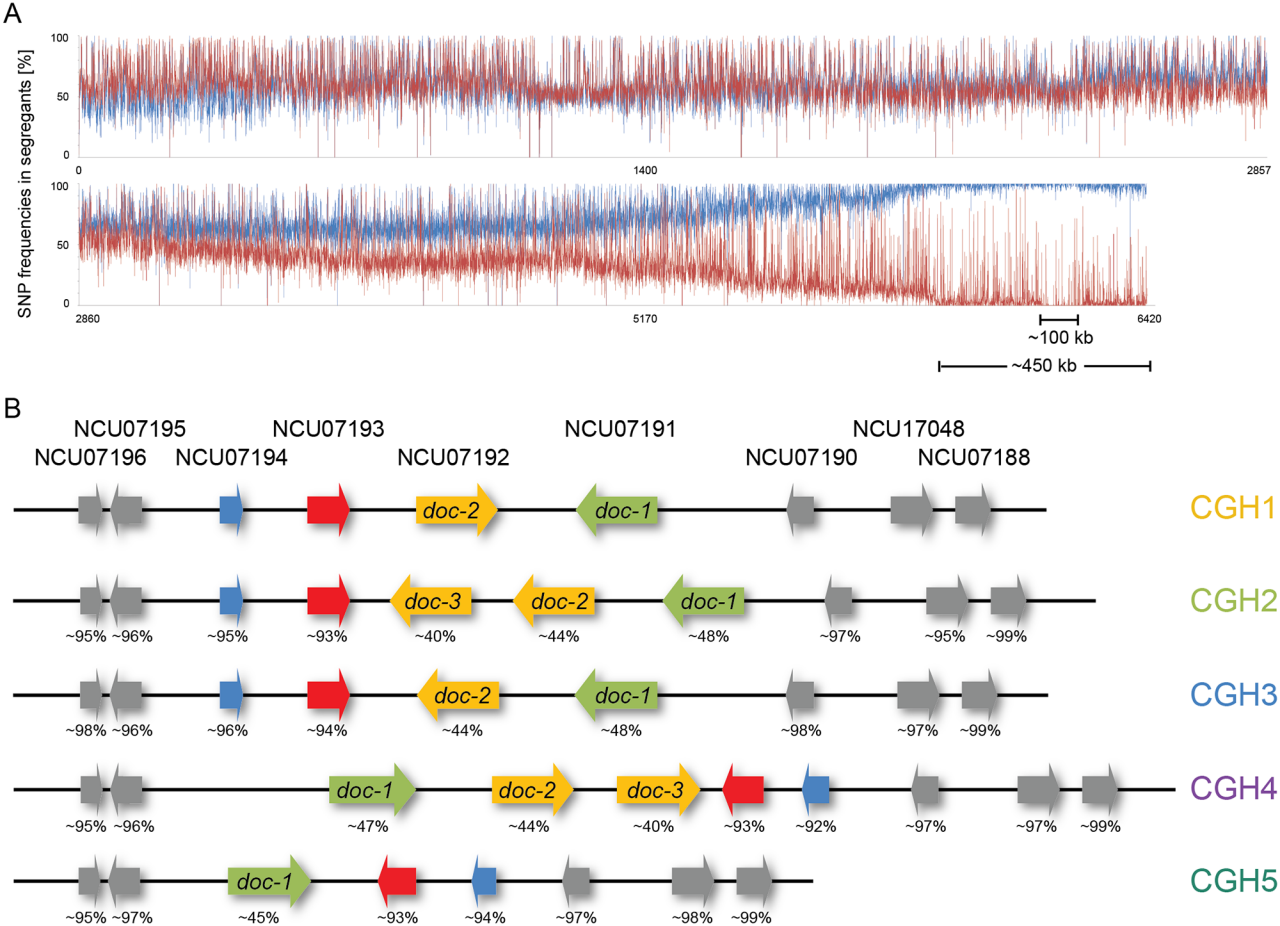
Communication group haplotypes. (A) SNP segregation on linkage group V after bulk segregant analyses and sequencing using pooled genomic DNA from 46 CG1 versus 46 CG2 progeny. Blue line: SNP frequency in pooled segregants that communicated with the CG1 tester. Red line: SNP frequencies in the pooled segregants that communicated with the CG2 tester. SNP frequencies are shown in comparison to the reference genome (FGSC 2489) [56]. (B) Genomic organization of communication group haplotypes (CGH) in wild isolates. Genomic rearrangements within the CGHs spanned the genetic interval between NCU07191 and NCU07194 and included duplications of NCU07192 (*doc-2*, *doc-3*), a deletion of NCU07192, and inversions. Alleles at NCU07191 (*doc-1*) and NCU07192 (*doc-2*, *doc-3*) within a CGH show high DNA sequence identity, but are polymorphic between CGHs. The percent DNA identity between alleles in members of the different CGH groups across the genetic interval in comparison to FGSC 2489 (a member of CGH1) are shown.

Based on the distribution of communication groups in the Louisiana population, we reasoned that genes that conferred kind recognition in *N*. *crassa* functioned as a Mendelian trait. To test this hypothesis, we used crossings to determine the number of loci mediating CG affiliation, making use of the fact that the affiliation of strains in the different CGs does not affect sexual compatibility [54]. We crossed a CG1 strain (FGSC 2489) with a CG2 strain (JW258), a CG1 strain (FGSC 2489) with a CG3 strain (D113), and a CG2 strain (JW242) with a CG3 strain (D113). In all crosses, the CG phenotype of the progeny segregated approximately 1:1, with approximately one-half of the progeny communicating with one parental strain and the second half of the progeny communicating with the other parent, consistent with our prediction that a single locus or closely linked loci were involved in kind recognition and determined CG affiliation. To identify the CG locus, we performed a bulk segregant analysis followed by whole genome resequencing of progeny from a cross between a CG1 strain (FGSC 2489) and a CG2 strain (JW258). Genomic DNA from 46 CG1 progeny or 46 CG2 progeny was isolated, pooled, and sequenced, revealing a ~100 kbp region on the right arm of linkage group V that showed segregation of SNPs between CG1 versus CG2 progeny at ~100% frequency, which was embedded within a larger divergent region of ~450 kbp (Fig 2A). A random SNP distribution of ~50% was observed for the remaining six linkage groups.

We used resequencing data from 26 wild isolates from the Louisiana population [55] to define allelic sequences at the CG locus within the ~100 kbp region. Among these 26 strains, seven isolates were members of CG1, 15 isolates were in CG2, and three isolates were members of CG3, while one isolate did not produce sufficient asexual spores to determine CG affiliation (asterisks in Fig 1C; S1 Table). Analysis of the ~100 kbp interval in the genomes of these 26 isolates revealed a 14 kbp region that showed five different genomic rearrangements that spanned four loci, NCU07191 to NCU07194 (gene nomenclature based on the reference genome from FGSC 2489 [56]), referred to as communication group haplotype 1 through 5 (CGH1–5) (Fig 2B). Of the 26 isolates, seven showed a CGH1 organization, five showed a CGH2 organization, three had a CGH3 organization, four had a CGH4 organization, and seven had a CGH5 organization. The CGH2 and CGH4 strains contained a duplication of NCU07192 (*doc-3*), while CGH5 strains did not contain NCU07192 (Fig 2B). Inversions of NCU07192 (CGH2 and CGH3 isolates) or the entire genetic interval between NCU07191 and NCU07194 were also observed (CGH4 and CGH5 isolates). Of the genes within this region, NCU07191 and NCU07192 showed ~43% DNA sequence identity, suggesting that they are paralogous. Paralogy was also supported by analyses using OrthoMCL (OG5_241519) [57].

To determine whether structural differences between CGHs were also associated with nucleotide differences, we used sequence alignments to characterize the nature and level of variability at genes within the haplogroups. Among five loci in the genetic interval associated with CGH, NCU07190, NCU07193, and NCU07194 displayed a high level of conservation among all 26 isolates (>90% DNA sequence identity with few nucleotide substitutions; Fig 2B; S2 Table). In contrast, NCU07191 and NCU07192 displayed high levels of allelic variability among the 26 isolates, with alleles falling into five main groups, which correlated with the genomic rearrangements among the five CGHs. Alleles at NCU07191 and NCU07192 showed only ~50% DNA sequence identity, with members of the different CGHs being highly divergent (0.22 to 0.74 differences/bp between NCU07191 alleles and 0.26 to 0.87 differences/bp between NCU07192 alleles; S2 Table). The predicted proteins encoded by NCU07191 and NCU07192 among members of the different CGH groups were also highly variable, with only ~35% amino acid identity (CGH1 versus all other CGH isolates), with few regions showing high conservation in all of the predicted NCU07191 or NCU07192 proteins (S2 Fig). In contrast, isolates within a single CGH showed DNA and amino acid identity at NCU07191 and NCU07192 that were comparable with the rest of the genome (up to 99% DNA and over 95% amino acid sequence identity) (S2 Fig), with the exception of the CGH1 isolates. Within the CGH1 isolates, alleles at NCU07191 and NCU07192 fell into two different subgroups (CGH1A and CGH1B), with ~70% DNA and ~60% amino acid sequence identity between members of the two subgroups (S2 Table). DNA sequence alignments of the genetic interval between NCU07191 and NCU07194 of the CGH1 isolates indicated that there were CGH1A- and CGH1B-specific indels and SNPs in the intergenic region between NCU07190 and NCU07193 (S3 Fig). Similarly, both CGH-specific SNPs resulting in amino acid substitutions and CG-specific indels differentiated isolates between the different CGH groups (S2 Fig).

The presence of five genomic CGHs with only three phenotypically distinguishable communication groups within the Louisiana population prompted us to reevaluate the communication phenotype of the 26 sequenced strains. For members of CG1 and CG3, the germling communication phenotype was completely correlated with CGH (S1 Table; Fig 2B); no difference in germling communication frequency was observed in isolates between the subgroups CGH1A and CGH1B. Unlike the CG1/CGH1 and CG3/CGH3 strains, the 15 strains defined as CG2 displayed multiple different genomic arrangements in this region (CGH2, CGH4, and CGH5; Fig 2B). However, using an isolate from each defined communication group (FGSC 2489, CG1; JW262, CG2; P4483, CG3), we identified a fourth phenotypic communication group (CG4). Germlings within this communication group (D111, JW179, P4479, P4489; S1 Table) showed very low communication frequencies with CG1 germlings, but underwent robust chemotropic interactions with both CG2 and CG3 germlings (S1 Table). These CG4 strains all showed the same genomic organization in the NCU07191 to NCU07194 genetic interval (CGH4), and with high DNA and amino acid sequence identity (>99%). Thus, of the 26 sequenced strains, seven isolates fell into CG1, with a CGH1 genomic organization; 11 isolates fell into CG2, with a CGH2 or CGH5 genomic organization; three isolates fell into CG3, with a CGH3 genomic organization; and four isolates were CG4 with a CGH4 genomic organization. An additional isolate (JW246) did not produce asexual spores, but had a CGH2 genomic organization.

We refer to NCU07191, NCU07192, and the duplicated version of NCU07192 as determinant of communication 1, 2, and 3 (*doc-1*, *doc-2*, and *doc-3*, respectively). *doc-1*, *doc-2*, and *doc-3* encode predicted hypothetical proteins (DOC-1, 828 aa; DOC-2, 839 aa; DOC-3, 920 aa). All of the DOC-2 proteins from the different CGH groups have a predicted OmpH-like outer membrane protein domain, although with some variability in conservation (S2 Fig), while DOC-1 and DOC-3 lack any identifiable functional domains. DOC-1 and DOC-2 contain one or two predicted transmembrane domains, while no transmembrane domain was predicted for DOC-3. Conserved homologs of *doc-1*, *doc-2*, and *doc-3* were identifiable by BLAST in the Sordariales (order within the class Sordariomycetes in the division Ascomycota), but were not obvious in more distantly related fungal species.

### *doc-1* and *doc-2* Determine Communication Group Affiliation

**Fig 3:**
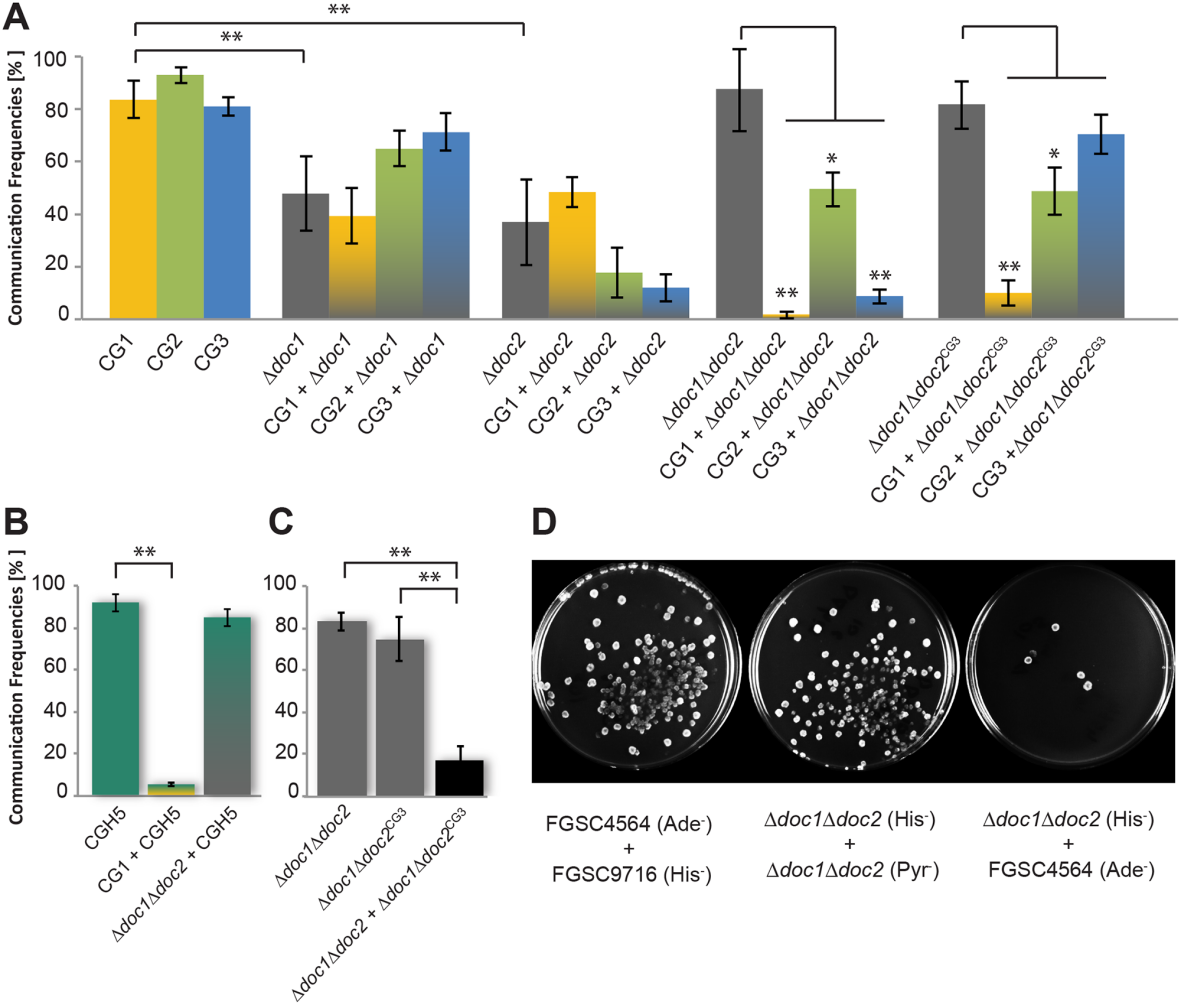
Communication interactions of Δ*doc-1*, Δ*doc-2*, *Δdoc-1 Δdoc-2*, and *Δdoc-1 Δdoc-2* (*his-3*::*doc-1* ^*CG3*^ *doc-2* ^*CG3*^) germlings. (A) Self-communication and non-self-communication frequencies between germlings of different mutants and the communication group tester strains. One-color bars denote self-communication frequencies between genetically identical germlings from a single strain, while two-color bars denote communication frequencies between genetically different germlings. *Δdoc1Δdoc2* ^*CG3*^ denotes *Δdoc-1 Δdoc-2* (*his-3*::*doc-1* ^*CG3*^ *doc-2* ^*CG3*^). CG tester strains were FGSC 2489, CG1; JW262, CG2; P4483, CG3. (B) Self-communication frequencies of CGH5 germlings (JW220) and non-self-communication frequencies of CGH5 germlings with CG1 (FGSC 2489) germlings (CG1 + CGH5), which show very low communication frequency. In contrast, *Δdoc-1 Δdoc-2* germlings (deletion strain derived from FGSC 2489) show high communication frequencies with a CGH5 strain (JW220) (*Δdoc-1 Δdoc-2* + CGH5). (C) Self-communication frequencies of *Δdoc-1 Δdoc-2* germlings (left grey bar), self-communication frequencies of *Δdoc-1 Δdoc-2* (*his-3*::*doc-1* ^*CG3*^ *doc-2* ^*CG3*^) germlings (right grey bar), and non-self-communication frequencies of *Δdoc-1 Δdoc-2* (*his-3*::*doc-1* ^*CG3*^ *doc-2* ^*CG3*^) germlings with *Δdoc-1 Δdoc-2* germlings (right bar). *Δdoc1Δdoc2* ^*CG3*^ denotes *Δdoc-1 Δdoc-2* (*his-3*::*doc-1* ^*CG3*^ *doc-2* ^*CG3*^). Experiments for A, B, and C were performed in triplicate with at least 100 germling pairs counted for each experiment. Black bars indicate standard deviations (Student's *t* test, *: *p* < 0.05, **: *p* < 0.01; see S1 Data for numerical values). (D) Quantitative heterokaryon test, in which conidia of CG1 strains with complementary auxotrophic markers (FGSC 4564; *ad-3B* + FGSC 9716; *his-3*), or conidia from *Δdoc-1 Δdoc-2* strains with complementary auxotrophic markers (*Δdoc-1 Δdoc-2; his-3* + *Δdoc-1 Δdoc-2; pyr-4)* or conidia from a CG1 strain and a *Δdoc-1 Δdoc-2* strain with complementary auxotrophic markers (FGSC 4564; *ad-3B* + *Δdoc-1 Δdoc-2; his-3*) were mixed and spread on modified VMM agar plates (see Materials and Methods for details). Colonies form only if germlings/hyphae containing complementary auxotrophic markers undergo fusion to form a prototrophic heterokaryon. Images show the results of one out of three experiments (see S4E Fig for quantitative data).

The association of communication group phenotype with CGH supported the hypothesis that the *doc* genes confer communication group specificity. To evaluate this hypothesis, we examined strains carrying deletions of *doc-1* or *doc-2* for communication phenotype. Strains carrying deletions of *doc-1* (*Δdoc-1*) or *doc-2* (*Δdoc-2*) in the CG1 background (FGSC 2489) [58] were macroscopically indistinguishable from FGSC 2489. To determine germling communication group phenotype in these deletion strains, we constructed *Δdoc-1* and *Δdoc-2* strains carrying a gene encoding cytoplasmic green fluorescent protein (GFP). Conidia of the communication group tester strains (FGSC 2489, JW262, or P4483) were stained with the membrane-selective endocytic dye FM4-64, mixed with the *Δdoc-1* (GFP) or *Δdoc-2* (GFP) strains, and subsequently analyzed for germling communication frequencies (Fig 3A). These analyses revealed that both the *Δdoc-1* and *Δdoc-2* germlings were impaired in self-communication: communication between isogenic *Δdoc-1* germlings was reduced to 48 ± 14%, as compared to the parental strain frequency of 84 ± 7%, while self-communication frequency in *Δdoc-2* germlings was reduced to 37 ± 16%.

Although self-communication frequency was reduced in *Δdoc-1* and *Δdoc-2* germlings, non-self-communication frequencies with germlings from the different communication group tester strains were similar to each other and to the self-communication frequencies of each deletion strain. For example, when *Δdoc-1* germlings were paired with either CG2 or CG3 germlings, non-self-communication frequencies were similar or even slightly higher than self-communication frequencies of *Δdoc-1* germlings (65 ± 7% for *Δdoc-1* + CG2 germlings; 71 ± 7% for *Δdoc-1* + CG3 germlings; Fig 3A). Similarly, non-self-communication frequencies of the *Δdoc-2* germlings with CG2 and CG3 tester strains were not significantly different from self-communication frequencies (Fig 3A), although communication frequencies of *Δdoc-2* germlings with the parental CG1 strain were higher (48 ± 6%) than with the CG2 (18 ± 9%) or CG3 tester strains (12 ± 5%). These data indicate that *doc-1* and *doc-2* are essential for mediating communication group discrimination in *N*. *crassa*.

### Germlings from a *Δdoc-1 Δdoc-2* Mutant Switch Communication Group

Since both the *Δdoc-1* and *Δdoc-2* mutants showed reduced germling communication frequencies, but no significant difference in the frequency of communication with members of the three communication groups, we hypothesized that a *Δdoc-1 Δdoc-2* double mutant would be completely deficient in communication and cell fusion. To test this hypothesis, we created a *Δdoc-1 Δdoc-2* mutant by homologous recombination (see Materials and Methods). As with the *Δdoc-1* or *Δdoc-2* single mutants, the *Δdoc-1 Δdoc-2* mutant was morphologically indistinguishable from its parental strain (FGSC 2489), a phenotype different than other fusion mutants, which display a “flat” phenotype [50]. To our surprise, unlike the single *Δdoc-1* or *Δdoc-2* mutants, the *Δdoc-1 Δdoc-2* mutant showed a self-communication frequency that was indistinguishable from its parental strain (~85%; Fig 3A). Even more surprisingly, the communication frequency between *Δdoc-1 Δdoc-2* germlings and their otherwise isogenic parental CG1 strain (FGSC 2489) was extremely low (~2%). The *Δdoc-1 Δdoc-2* germlings also showed very low communication frequency with the CG3 tester strain (P4483, ~8%; Fig 3A). In contrast, the *Δdoc-1 Δdoc-2* germlings communicated fairly well with the CG2 tester strain (JW262, ~50%), although still significantly less than self-communication frequencies of *Δdoc-1 Δdoc-2* germlings.

The communication phenotype of the *Δdoc-1 Δdoc-2* germlings suggested that DOC-1 /DOC-2 negatively regulate germling communication behavior and that removal of DOC-1/DOC-2 resulted in the generation of a new communication group. We further tested this hypothesis by evaluating the communication phenotype of *Δdoc-1 Δdoc-2* germlings with additional members of CGH2, CGH3, CGH4, and CGH5. A CGH3 (D113) strain and CGH4 strains (D111, P4489) showed very low communication frequencies with *Δdoc-1 Δdoc-2* germlings (~10%), while CGH2 strains (JW258, P4463) showed a reduction in communication frequency with *Δdoc-1 Δdoc-2* germlings, as was observed for the CG2 tester strain JW262 (~50%; S4 Fig). However, in contrast to members of the other CGH groups, germling communication frequencies of the *Δdoc-1 Δdoc-2* mutant with members of CGH5 (JW75, JW220, and JW242) were identical to self-communication frequencies (Fig 3B; S4 Fig). These data showed that the *Δdoc-1 Δdoc-2* mutant behaved exactly like members of CGH5, which contained one copy of *doc-1* but no copy of *doc-2*. Thus, the phenotype of the *Δdoc-1 Δdoc-2* mutant with the CGH5 isolates defined a new communication group, termed CG5.

The availability of isogenic strains that only differed in communication behavior (FGSC 2489, CG1; *Δdoc-1 Δdoc-2*, CG5) allowed us to assess whether just the genetic difference at *doc-1* and *doc-2* was sufficient to affect the formation of heterokaryons (i.e., a syncytium of two or more genetically different nuclei), which is mediated by both germling and hyphal fusion. We introduced auxotrophic markers (*his-3* or *pyr-4*) into a *Δdoc-1 Δdoc-2* strain and first evaluated its ability to form (*Δdoc-1 Δdoc-2; his-3* + *Δdoc-1 Δdoc-2; pyr-4*) heterokaryotic colonies as compared to strains isogenic to their parent (FGSC 2489) but that carry complementary auxotrophic markers (*his-3* or *ad-3B)* (S4 Table). As shown in Fig 3D, heterokaryon formation was indistinguishable between (*Δdoc-1 Δdoc-2; his-3* + *Δdoc-1 Δdoc-2; pyr-4*) strains and (*his-3* + *ad-3B*) strains. In contrast, heterokaryon formation was drastically reduced when *Δdoc-1 Δdoc-2; his-3* conidia were mixed with *ad-3B* conidia (Figs 3D and S4E). These data indicated that even if communication and cell fusion were essential for survival of *N*. *crassa*, differences in communication group affiliation almost completely prevented cooperation via heterokaryon formation.

### *doc-1* and *doc-2* Are Sufficient for Communication Group Affiliation

To determine if *doc-1* and *doc-2* were sufficient for communication group affiliation, we cloned the *doc-1* and *doc-2* alleles from a CG3 strain (P4471; *doc-1* ^*CG3*^ and *doc-2* ^*CG3*^) and targeted them to the *his-3* locus in the *Δdoc-1 Δdoc-2* mutant. The *Δdoc-1 Δdoc-2* (*his-3*::*doc-1* ^*CG3*^ *doc-2* ^*CG3*^ *)* strain was macroscopically indistinguishable from the laboratory strain from which it was derived (FGSC 2489). Germlings from *Δdoc-1 Δdoc-2* (*his-3*::*doc-1* ^*CG3*^ *doc-2* ^*CG3*^ *)* showed high self-communication frequencies (~80%; Fig 3A and 3C). However, *Δdoc-1 Δdoc-2* (*his-3*::*doc-1* ^*CG3*^ *doc-2* ^*CG3*^ *)* germlings showed greatly reduced communication frequency with their parental *Δdoc-1 Δdoc-2* strain, showing that *doc-1* ^*CG3*^ and *doc-2* ^*CG3*^ were functional in this strain (Fig 3C). Germlings from the *Δdoc-1 Δdoc-2 (his-3*::*doc-1* ^*CG3*^ *doc-2* ^*CG3*^ *)* strain also showed low communication frequency with the CG1 tester strain (FGSC 2489), but showed some communication with the CG2 tester strain (JW262), although germling communication frequencies were reduced (~50%; Fig 3A). However, the *Δdoc-1 Δdoc-2 (his-3*::*doc-1* ^*CG3*^ *doc-2* ^*CG3*^ *)* germlings communicated well with the CG3 tester strain (P4483) and the donor for *doc-1* ^*CG3*^ and *doc-2* ^*CG3*^ alleles (P4471) (Figs 3A and S4D). These data indicated that addition of *doc-1* ^*CG3*^ and *doc-2* ^*CG3*^ to the *Δdoc-1 Δdoc-2* mutant was sufficient to switch communication group from CG5 to CG3. To confirm that the *Δdoc-1 Δdoc-2 (his-3*::*doc-1* ^*CG3*^ *doc-2* ^*CG3*^ *)* germlings no longer belonged to CG5, we tested communication frequencies of *Δdoc-1 Δdoc-2 (his-3*::*doc-1* ^*CG3*^ *doc-2* ^*CG3*^ *)* germlings with a CG5 strain (JW242). In contrast to *Δdoc-1 Δdoc-2* germlings, *Δdoc-1 Δdoc-2 (his-3*::*doc-1* ^*CG3*^ *doc-2* ^*CG3*^ *)* germlings showed low communication frequency with JW242 germlings (~15%; S4C Fig versus S4D Fig). Thus, swapping *doc-1* and *doc-2* alleles from a member of CGH1 with *doc-1* and *doc-2* alleles from a member of CGH3 was sufficient to switch communication group.

### DOC-1 Oscillates to the Tips of Conidial Anastomosis Tubes and Fusion Hyphae during Chemotropic Interactions

**Fig 4:**
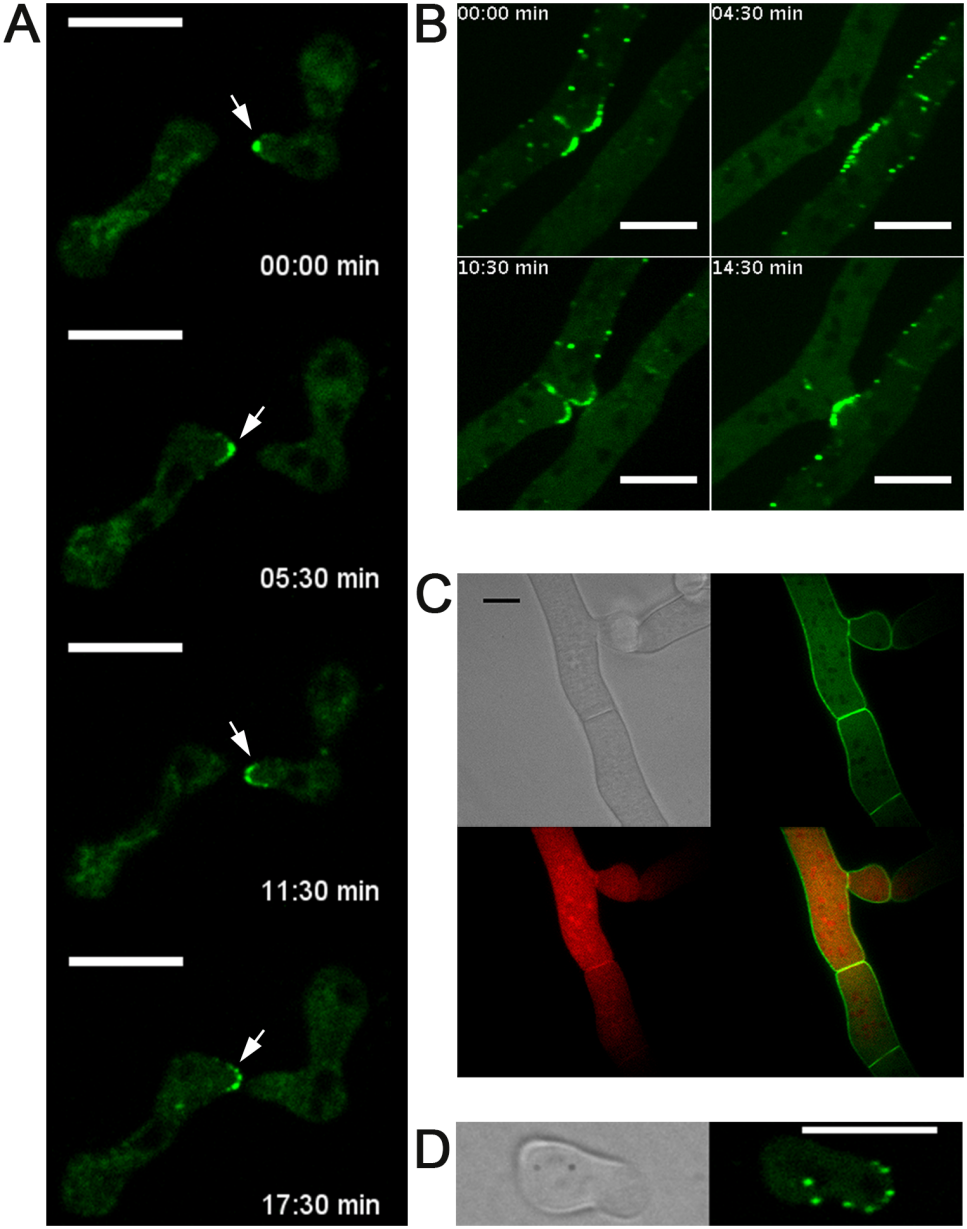
Cellular localization of DOC-1-GFP and DOC-2-GFP. (A) DOC-1-GFP showed dynamic localization to puncta at the tips of conidial anastomosis tubes during chemotropic interactions between genetically identical cells, with an oscillation period of 8–10 min within a single germling tip. See S5B Fig for oscillation intervals and S3 Movie for interacting germlings. (B) Oscillation of DOC-1-GFP in hyphae in a single colony undergoing chemotropic interactions prior to cell fusion. See Fig 5A for oscillation intervals and S4 Movie for interacting fusion hyphae. (C) DOC-2-GFP (green, top right panel) localized to the plasma membrane in mature hyphae. Co-localization between DOC-2-GFP with MAK-2-mCherry (red, bottom left panel) was not observed (overlay, bottom right panel). (D) *tef-1* driven GFP-DOC-2 (green) showed localization to puncta in germlings, but that do not show oscillation during chemotropic interactions. Scale bars: 10 μm.

**Fig 5:**
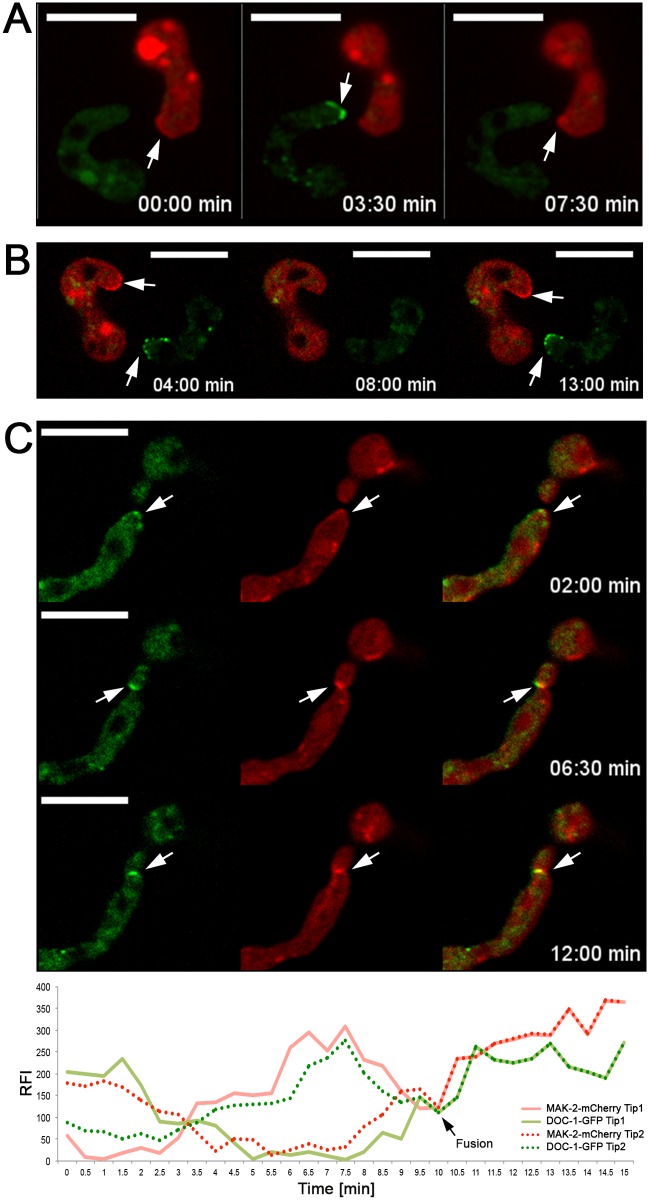
DOC-1-GFP co-oscillates with MAK-2 during chemotropic interactions. (A) Germlings expressing DOC-1-GFP (green) were paired with germlings expressing MAK-2-mCherry (red). When MAK-2-mCherry accumulated at one CAT tip (arrows left and right panel), DOC-1-GFP was absent from the tip of the interacting germling. When DOC-1-GFP accumulated at the second CAT tip (arrow middle panel), MAK-2-mCherry was absent from the first CAT tip, indicating that DOC-1-GFP showed identical oscillation dynamics to MAK-2-mCherry. (B) Germlings expressing DOC-1-GFP (green) were paired with germlings expressing SOFT-mCherry (red). When SOFT-mCherry accumulated at one CAT tip, DOC-1-GFP accumulated at the CAT tip of its interacting partner (arrows left and right panel). When SOFT-mCherry was absent from the first CAT tip, DOC-1-GFP was absent from the second CAT tip (middle panel), indicating that DOC-1-GFP showed opposite oscillation dynamics to SOFT-mCherry. See S5B Fig for oscillation intervals. (C) Co-localization and co-oscillation of DOC-1-GFP (left panel) with MAK-2-mCherry (middle panel) in heterokaryotic germlings undergoing chemotropic interactions (overlay, right panel). Bottom panel: Graphic representation of DOC-1-GFP and MAK-2-mCherry signals at the tips of conidial anastomosis tubes in germlings undergoing chemotropic interactions. *y*-axis shows the ratio of relative fluorescence intensity (RFI) in the interacting zone as compared to background. *x*-axis shows time (min). Note the co-oscillation of both DOC-1-GFP and MAK-2-mCherry in both germlings following fusion, as shown previously for MAK-2 and SOFT [45].

Self-recognition between isogenic germlings requires the MAK-2 MAP kinase complex (NRC-1, MEK-2, MAK-2, and the scaffold protein HAM-5), which oscillates to the tips of conidial anastomosis tubes and completely out-of-phase with SOFT, a scaffold protein for the cell wall integrity MAPK pathway (Fig 1A) [45–47,59,60]. To determine the cellular location of DOC-1 and DOC-2 during germling communication, and its relationship with temporal patterns of signaling, we constructed strains bearing *doc-1-gfp* and *doc-2-gfp* alleles that were fully functional in restoring communication frequencies in Δ*doc-1* or Δ*doc-2* germlings, respectively (S4 and S5 Figs). In self pairings between Δ*doc-1* (*doc-1-gfp*) germlings, DOC-1-GFP localized to intracellular punctae, which oscillated to the tips of conidial anastomosis tubes during chemotropic interactions (Fig 4A), with an interval of 8–10 min (S3 Movie; S5 Fig), an identical oscillation pattern to that of the MAK-2 complex and SOFT [44]. To investigate whether DOC-1 oscillates with MAK-2 or with SOFT during chemotropic interactions, we analyzed DOC-1-GFP oscillation in germlings undergoing chemotropic interactions with germlings bearing MAK-2-mCherry or SOFT-mCherry; DOC-1 oscillated with MAK-2, but completely out of phase to SOFT (Figs 5A, 5B and S5; S3 Movie). A heterokaryotic strain bearing both DOC-1-GFP and MAK-2-mCherry confirmed these observations (Fig 5C).

In hyphae, DOC-1-GFP localized to puncta that also oscillated at the hyphal tip during chemotropic interactions prior to hyphal fusion (similar to MAK-2 and HAM-5; [45]), as well as to septa (Fig 4B; S4 Movie). To determine whether DOC-1-GFP localization was dependent on MAK-2, we expressed DOC-1-GFP in a strain deleted for *mak-2*. The Δ*mak-2* (*doc-1-gfp*) strain showed a typical Δ*mak-2* phenotype, including lack of chemotropic interactions and cell fusion. Additionally, DOC-1-GFP showed cytoplasmic and vacuolar localization and never localized to puncta, although localization to septa was retained in this strain. Thus, DOC-1 is a component of the MAK-2 signaling complex and co-oscillates with this complex during chemotropic interactions.

A *doc-2-gfp* allele regulated by the *ccg-1* promoter localized to the plasma membrane and septa in mature colonies (Fig 4C). When a fully functional *gfp-doc-2* allele was placed under the regulation of the *tef-1* promoter (*ccg-1* has low expression levels in germlings compared to *tef-1*; [47]), GFP-DOC-2 localized to puncta in germlings (Fig 4D). However, oscillation of DOC-2 during chemotropic interactions in either germlings or in fusion hyphae was never observed.

### DOC-1/DOC-2 Mediate Communication Group Discrimination by Preventing Reinforcement of Signaling

**Fig 6:**
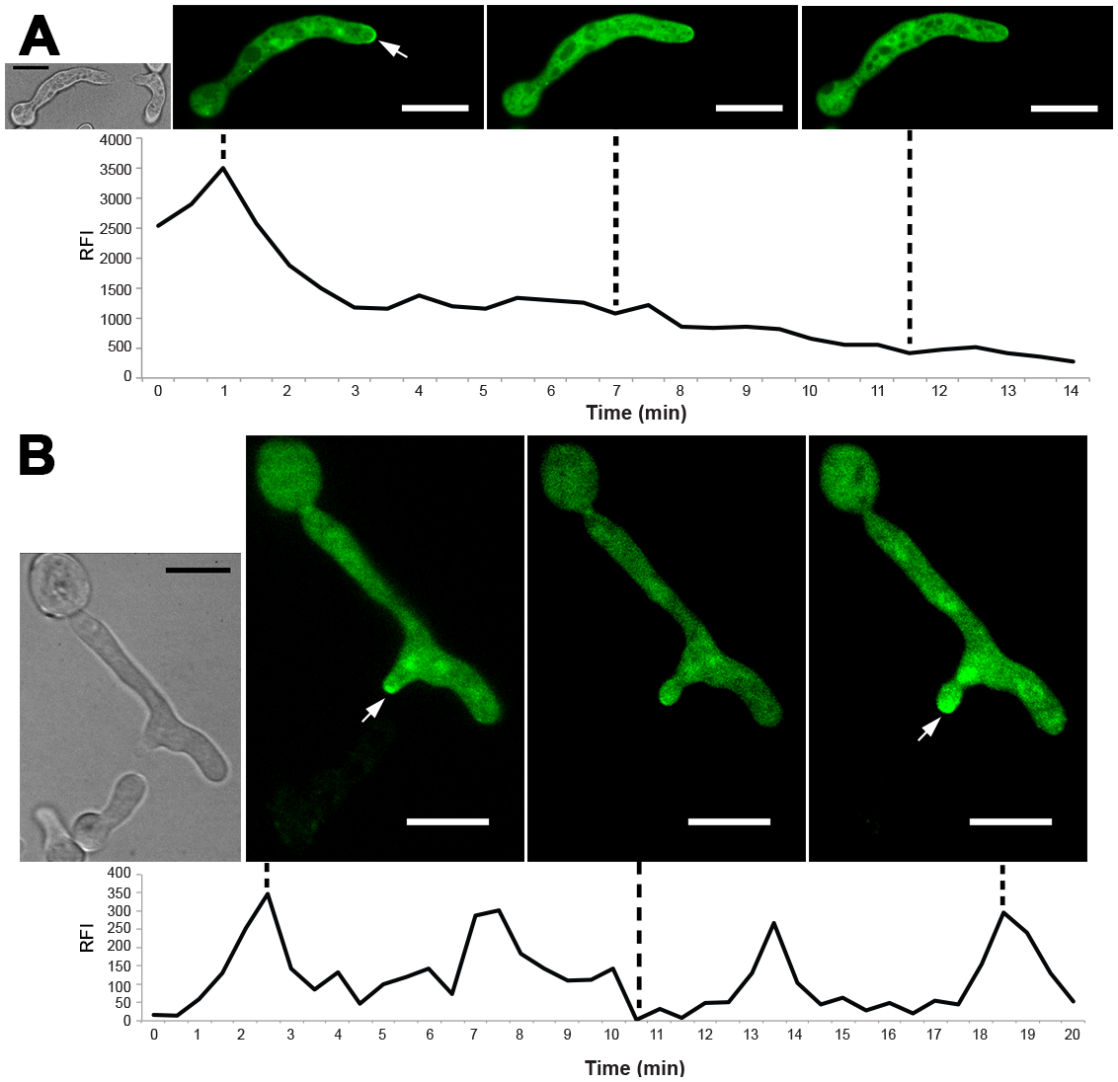
Enforcement of oscillation of signaling proteins is necessary to establish chemotropic interactions regardless of CG. (A) MAK-2-GFP localized to the tips of conidial anastomosis tubes in a *Δdoc-1 Δdoc-2* (CG5) germling when in proximity to a CG2 (JW262) germling (top panel). However, further oscillation of MAK-2-GFP did not occur, and chemotropic interactions between the *Δdoc-1 Δdoc-2* (CG5) germling and JW262 (CG2) germling were not established. Bottom panel: Graphic representation of MAK-2-GFP signals at the tip of a *Δdoc-1 Δdoc-2* (CG5) germling in proximity to a JW262 (CG2) germling. *y*-axis shows the ratio of relative fluorescence intensity (RFI) in the interacting zone as compared to background. *X*-axis shows time (min). (B) MAK-2-GFP in a *Δdoc-1 Δdoc-2* germling shows continued oscillation to the CAT tip if chemotropic interactions are established between *Δdoc-1 Δdoc-2* (CG5) and JW262 (CG2) germlings (top panel). Bottom panel: Graphic representation of MAK-2-GFP signal at the CAT tip of *Δdoc-1 Δdoc-2* (CH5) germlings undergoing chemotropic interactions with germlings of JW262 (CG2). *y*-axis shows the ratio of RFI in the interacting zone as compared to background. *x*-axis shows time (min) (See S5 Movie for second example).

The oscillation of signaling components is necessary to maintain chemotropic interactions; inhibition of MAK-2 kinase activity obliterated oscillation and chemotropic interactions in communicating germlings [44]. The function of DOC-1/DOC-2 in communication group discrimination could be to prevent initiation of signaling, or to prevent reinforcement of signaling, which is hypothesized to be required for sustained chemotropic interactions [49,61]. To differentiate between these two hypotheses, we analyzed localization of MAK-2-GFP in *Δdoc-1 Δdoc-2* germlings when they were in close proximity to CG2 germlings (JW262), where communication frequency is ~50% (Fig 3A). Prior to chemotropic interactions, localization of MAK-2-GFP in *Δdoc-1 Δdoc-2* germlings was observed at the tip when in close proximity to CG2 germlings (Fig 6A). However, chemotropic interactions were only established if oscillation of MAK-2-GFP occurred (Fig 6B; S5 Movie). If oscillation of MAK-2 was not maintained, chemotropic interactions between *Δdoc-1 Δdoc-2* and CG2 germlings were abolished. These observations suggest that DOC-1/DOC-2 do not function at the recognition stage of germling interactions, but instead function to mediate communication group discrimination at the point where robust oscillation of the MAK-2 signaling complex to the tips of conidial anastomosis tubes is reinforced, and which is essential for further chemotropic interactions.

The necessity of coordinated and out-of-phase oscillation of MAK-2 complex with SOFT for successful communication [44] suggested that the DOC proteins might influence the oscillation interval (8–10 min in CG1 strains) and that differences in oscillation timing might determine communication group affiliation. To test this hypothesis, we compared the oscillation timing of SOFT-GFP during chemotropic interactions in germlings from a CG1 strain (FGSC 2489 [*so-gfp*]) [44] as compared to the CG5 strain (*Δdoc-1 Δdoc-2* [*so-gfp*]). We quantified fluorescence intensities at the tip of communicating germlings and measured the interval between two fluorescence maxima. However, no significant differences between the oscillation intervals of SOFT could be detected for CG1 versus CG5 germlings, suggesting that alteration of oscillation timing was not the basis of communication group phenotype (S5 Fig).

### *doc-1*, *doc-2*, and *doc-3* Show Evidence of Long-Term Balancing Selection

**Fig 7:**
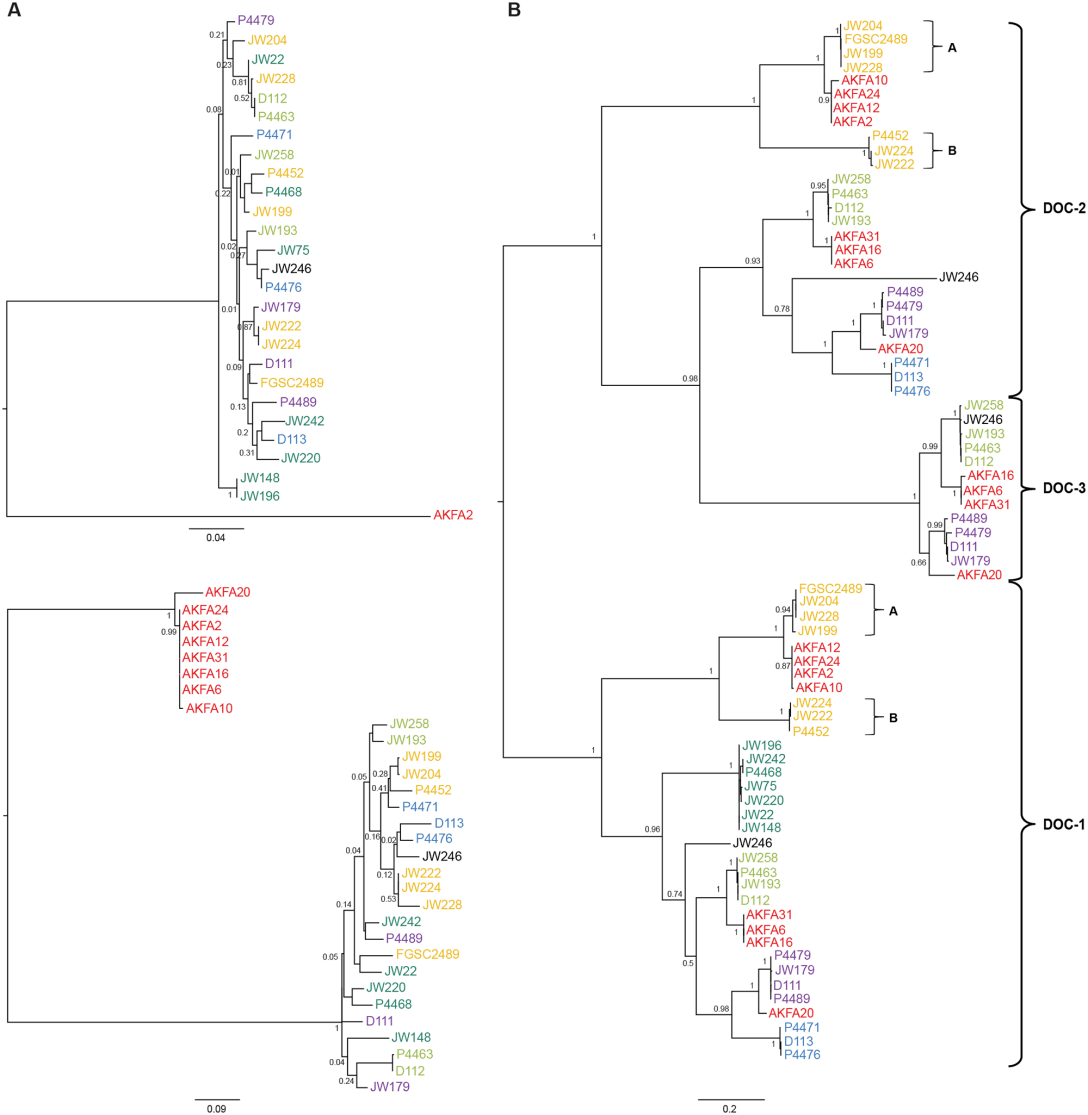
Phylogenetic analyses of the predicted proteins encoded by genes associated with genomic rearrangements revealed transspecies polymorphisms at DOC-1 and DOC-2/DOC-3. Coding sequences from 26 *N*. *crassa* wild Louisiana isolates and up to eight *N*. *discreta* wild isolates were used to build maximum likelihood phylogenetic trees for (A) NCU07190 (top) and NCU07193 (bottom) and (B) NCU07191 (*doc-1*) and NCU07192 (*doc-2*; *doc-3*), using the default pipeline from Phylogeny.fr [99]. Bootstrap values are given for each node. Black bars indicate substitution rates. CGH1 isolates are shown in orange (subgroups A and B are indicated), CGH2 isolates are shown in light green, CGH3 isolates are shown in blue, CGH4 isolates are shown in purple, and CGH5 isolates are shown in dark green. The isolate JW246 did not produce spores and is therefore shown in black. *N*. *discreta* isolates are shown in red. Note nesting of *N*. *discreta* isolates within *N*. *crassa* lineages for NCU07191 (*doc-1*) and NCU07192 (*doc-2*; *doc-3*), but not for NCU07190 or NCU07193 (shown in panel A); see S1 Tree for Nexus file.

Our data indicate that *doc-1* and *doc-2* function as helping greenbeard genes, with multiple alleles mediating assortative kind recognition by changing chemotropic behavior by negatively regulating interactions during germling fusion. The finding of five communication groups mediated by five highly divergent haplotypes suggested a relatively ancient origin of the communication locus controlling germling fusion. To test this hypothesis, we first performed phylogenetic analyses of alleles at NCU07190 through NCU07193 in the 26 sequenced wild isolates, as well as alleles at these same loci from a population sample from the distantly related *s*pecies *Neurospora discreta*. For NCU07190 and NCU07193, allelic lines from within species were reciprocally monophyletic (Fig 7A), as predicted by theory [62], given the estimated divergence time between *N*. *crassa* and *N*. *discreta* (7–10 million years ago [63]) and their effective population size (circa 10^6^ and 10^4^ individuals, respectively [51,64]). However, for the three *doc* genes, no reciprocal monophyly was observed, and *N*. *crassa* alleles from the same CGH-associated clade were closer to *N*. *discreta* alleles than to *N*. *crassa* alleles from another clade, indicating that the age of allelic lines exceeds the age of speciation events—a phenomenon referred to as transspecies polymorphism (Fig 7B). Inferred genealogical histories of *doc* genes were in fact concordant with differences in patterns of genomic arrangements among communication group haplotypes: alleles from CGH1 to CGH5 (including CGH1A and CGH1B) were in distinct clades for *doc-1*, and similar topologies were also inferred at *doc-2* (although CGH5 strains lack *doc-2*) and *doc-3* (found only in CGH2 and CGH4 strains; Figs 2 and 7B).

Transspecies polymorphism is a signature of long-term balancing selection [65], and evidence for balancing selection was also provided by tests of the standard neutral model using Tajima’s D, which measures skewness of the allele frequency spectrum (S3 Table). Tajima’s D values at NCU17048, NCU07190, NCU07193, or NCU07194 did not deviate from neutral expectations (Tajima’s D < 1; *p* > 0.1), while values of Tajima’s D were high, positive, and significant for *doc-1*, *doc-2*, and *doc*-3 (Tajima’s D > 2; *p* < 0.01 for *doc-1* and *doc-2*; *p* < 0.05 for *doc-3*). These data indicate that balancing selection is acting to maintain polymorphisms at *doc-1*, *doc-2*, and *doc-3*, but its signature is not detectable on surrounding genes.

The finding of long divergent haplotypes under balancing selection at the *doc* communication locus suggested that recombination rates might be reduced across the region, thereby preventing the migration of variants between allelic lines [65,66]. To test for recombination within the *doc* region between isolates of different CGHs, we analyzed concordance among genealogies of all genes within the region [67]. These analyses of genealogical concordance within the *doc* region revealed congruent branching of sequence groups from different CGHs for *doc-1*, *doc-2*, and *doc-3* over the entire length of the genes, consistent with a lack of recombination between haplotypes from different communication groups (S6 Fig). In contrast, an analysis of genealogical concordance within the *doc* region among haplotypes defining the same communication group was consistent with multiple recombination events (S7 Fig), except for CGH1 isolates, in which recombination was not observed within the *doc-1/2* region between the CGH1A and CGH1B isolates (S6 and S7 Figs). These data suggest that the recombination rate between haplotypes from different communication groups was reduced, probably because of strong selection against recombinants.

## Discussion

Frequency-dependent effects, involving the expression of traits with differential effects on bearers and nonbearers, are common in microbes and can be interpreted as kind discrimination via greenbeard genes [3,9]. Previously, microbial kind discrimination has been described as a post-contact process; for example, cell adhesion proteins in *D*. *discoideum* [7,14] or in *S*. *cerevisiae* [15]. Here, we show that the filamentous fungus *N*. *crassa* uses kind discrimination that acts at a distance to differentiate communication groups in wild populations. We show that this kind discrimination system is controlled by the paralogous greenbeard genes *doc-1*, *doc-2*, and *doc-3*, which together determine communication group affiliation. In genetically identical cells, chemotropic interactions are associated with the out-of-phase oscillation of MAK-2 and SOFT complexes [44–47], which is postulated to allow genetically identical and developmentally equivalent cells to coordinate their behavior while avoiding self-stimulation [22,44,49,68]; DOC-1 oscillates with MAK-2 during chemotropic interactions. Thus, kind discrimination mediated by the DOC proteins adds another layer of complexity to germling communication, because cells must not only avoid self-stimulation but also stimulation by non-kind individuals.

**Fig 8:**
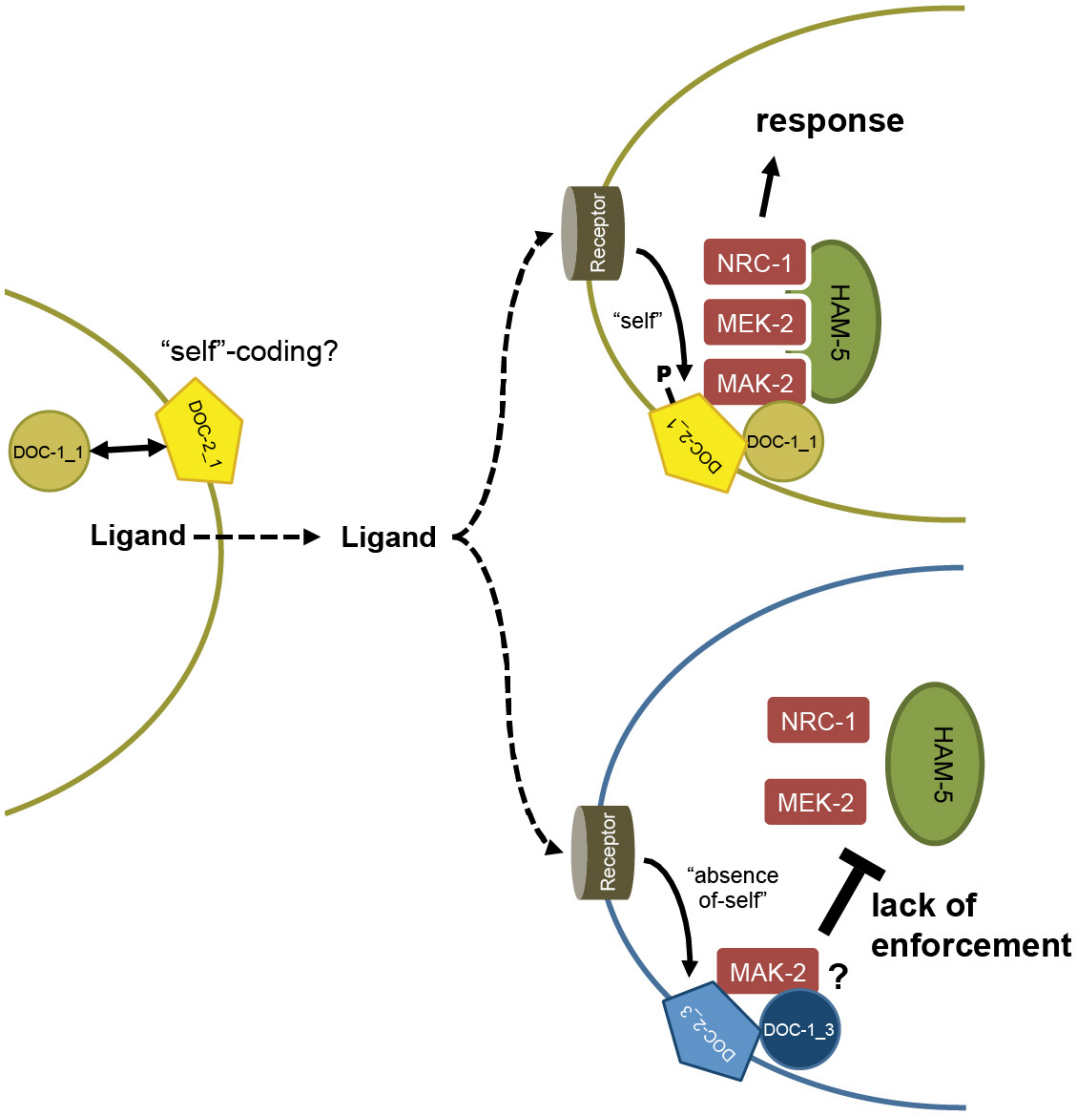
Model for DOC-1 and DOC-2 function in long-distance kind discrimination. This model assumes that during conidial germination, *N*. *crassa* germlings release a ligand that acts as a signal for potential interaction partners. The ligand/receptor or other components of the recognition pathway might be modified and coded as “self” by DOC-1/DOC-2 of the sending cell. In the receiving cell, the ligand activates a receptor. The DOC-1/DOC-2 system of the receiving cell functions in quality control. If the signal passes quality control, oscillation of the assembled MAK-2 complex is enforced and the signal-receiving cell shows chemotropic interactions (top). If the signal does not pass quality control, the DOC-1/DOC-2 system of the receiving cell prevents enforcement of MAK-2 oscillation, and, therefore, the receiving cell does not respond to the presence of a potential fusion partner (bottom).

Although ligand(s) and receptor(s) must exist to account for chemotropic interactions between fungal germlings and hyphae, screens of the *N*. *crassa* near full genome deletion set have failed to identify genes encoding these components [50,61,68]. Our working model incorporates a communication ligand/receptor, which serves as a universal signal for chemotropic interactions in this species (Fig 8). The communication receptor is activated in the receiving cell upon interaction with the ligand. This signal is transmitted intracellularly to DOC-1/DOC-2, which together function in quality control, an element commonly required for self-/non-self-discrimination [69]. Since *Δdoc-1 Δdoc-2* germlings undergo self-communication and chemotropic interactions, DOC-1 and DOC-2 must function to repress MAK-2 oscillation reinforcement if non-kind germlings are in close proximity, rather than being required for the activation of signaling. Chemotropic interactions between two germlings is established if quality control allows the reinforcement of the MAK-2 and SOFT oscillation rhythm in communicating germlings, even if they are of different communication groups. If the signal does not pass the quality control, reinforcement of MAK-2 oscillation is suppressed and chemotropic growth fails to occur.

The model for long-distance kind recognition in *N*. *crassa* is reminiscent of the “missing-self” theory for vertebrate natural killer cells and for non-self-recognition in the basal chordate, *Botryllus schlosseri* [70,71]. Instead of directly recognizing different non-self signals, anything that is not recognized as self by default is considered non-self. For natural killer cells and for the self-ligand *fuhc* with its effector system *fester* in *B*. *schlosseri*, a self-education process is predicted to occur that helps cells to adapt to the correct combination of cell surface receptors [72,73]. We predict that a similar process is mediated by DOC-1/DOC-2 to “educate” unknown recognition components involved in the reinforcement of MAK-2 signaling complex oscillation to the tips of conidial anastomosis tubes and fusion hyphae during chemotropic interactions. For example, membrane-bound protein DOC-2 may mediate kind signaling (“self”-coding), perhaps via modification/interaction with the receptor or ligand or other components involved in recognition (Fig 8).

In filamentous fungi, multiple loci confer self-/non-self-discriminations that act post-fusion and are typically among the most polymorphic loci in fungal genomes, but with a limited number of compatible allelic classes per locus [42,55,66,74–76]. Our studies revealed the existence of a single region of linked paralogous loci that confers at least five communication groups that function in self-/non-self-recognition during chemotropic interactions, prior to cell contact. CGH1 isolates additionally fell into two subgroups that showed divergent *doc-1* and *doc-2* alleles, which suggests that members of CGH1A and CGH1B groups may represent an additional sixth communication group. Although the presence of multiple long-diverged allelic lines is observed at fungal self-/non-self-discrimination loci, assortative kind recognition is not theoretically expected from kin selection theory. Indeed, if fusion between individuals is mutually beneficial (and/or rejection costly), individuals carrying a common recognition allele will more readily fuse and, hence, have a higher fitness than individuals carrying less frequent alleles. Hence, as its frequency increases, the recognition should be turned into a “greenbeard gene” that recognizes copies of itself and is being recognized by copies of itself, and it should reach fixation through positive-frequency-dependent selection, thereby removing the variation necessary to allow discrimination in the first place [3,12,16,77]. However, the finding of long-diverged alleles and transspecies polymorphism consistent with long-term balancing selection at germling fusion loci (this study) and previously characterized self-/non-self-discrimination loci acting post-fusion [36–38], suggests that additional extrinsic selective forces may promote the establishment and maintenance of assortative kind discrimination. For instance, self-/non-self-discrimination genes may directly experience balancing selection if kind recognition genes are maintained polymorphic by pathogen selection pressures causing rare allele advantage [18,39,78]. Bioinformatics and comparative genomics should help determine whether self-/non-self-discrimination genes such as *doc* genes have secondary functions that keep them variable.

Although kind discrimination mediated during cell contact has been described in organisms ranging from bacteria to colonial ascidians [38,71,79,80], the proteins and signals involved here are quite different. For invertebrates, it has been postulated that proteins controlling non-self-recognition are unique to each phylum [81]. It is possible that the core of non-self-recognition resides in intracellular conserved processes that integrate and respond to polymorphic external stimuli. We believe that kind discrimination mechanisms function in many filamentous fungi that are capable of undergoing cell/hyphal fusion. Hyphal avoidance has been described in a number of fungal species that are very distantly related to *N*. *crassa* [82,83], making filamentous fungi excellent models for investigating kind recognition mechanisms. Our study provides the basis for research in self-/non-self-recognition that will be applicable to attraction, fusion, and kind discrimination in other eukaryotic species.

## Material and Methods

### Strains and Growth Conditions

Standard protocols for *N*. *crassa* can be found on the *Neurospora* homepage at the Fungal Genetics Stock Center (FGSC, http://www.fgsc.net/Neurospora/NeurosporaProtocolGuide.htm). Strains were grown on Vogel’s minimal medium (VMM [84], with supplements as required) or on Westergaard’s synthetic cross medium for mating [85]. The wild *N*. *crassa* strains used in this study (S1 Table) were isolated from Louisiana, US and are available at the FGSC [52,86,87]. Manipulated strains are listed in S4 Table. FGSC 2489 served as parental strain for gene deletions and as a WT-control for all experiments, unless stated otherwise. Single deletion mutants are available at the FGSC [58,88]. The Δ*doc-2* mutant deposited at the *Neurospora* knockout collection showed a flat phenotype, and its conidia had slow germination rates. In a back cross with FGSC 2489, none of the phenotypes co-segregated with hygromycin resistance, indicating that it was due to a secondary mutation. To create the Δ*doc-1* Δ*doc-2* mutant, a deletion construct was created using the method of fusion PCR [89]. Briefly, ~1 kb of the 5′ regions of *doc-1* and *doc-2* was amplified by PCR from genomic DNA (S5 Table), and the hygromycin cassette was amplified from the vector pCSN44 [58]. The three fragments were fused in a fusion PCR reaction to create the deletion construct, which was used to transform the *Δmus-51* strain of *N*. *crassa* [58]. Hygromycin-resistant transformants were analyzed by PCR, and positive strains were backcrossed to FGSC 2489.

Histidine auxotrophic strains for complementation experiments were obtained by crossing the *doc* deletion strains with FGSC 6103. The plasmid pMF272 (AY598428) was modified to create *gfp*-fusions to *doc-1* and *doc-2*, which were targeted to the *his-3* locus [90]. A 300 bp fragment of the 3′ region of *ccg-1* was cloned 3′ of *gfp* open reading frame as a termination signal using the *Eco*RI restriction site. Plasmid derivatives with the *tef-1* promoter or native promoters were obtained by swapping out the *ccg-1* promoter using the restriction enzymes *Not*I and *Xba*I. For CG switch experiments, *doc-1* and *doc-2*, including their native promoter and terminator sequences, were amplified from genomic DNA of the isolate P4471 (S5 Table). Using the Gibson assembly, both fragments were cloned into the *Eco*RI/*Not*I digested vector pMF272 [91]. All constructs were transformed into FGSC 6103 with selection for His^+^ prototrophy and then crossed into the *doc* double or single deletion mutants.

### Assays for Germling Communication

Each strain was grown on VMM in slant tubes for 4–6 d or until significant conidiation occurred. Conidia were prepared by filtering 600 μl of conidial suspension through cheesecloth. An aliquot of 180 μl of a conidial suspension from one strain was mixed with 20 μl of FM4-64 solution (16 μM), incubated for 15 min, and subsequently washed with 1 ml of ddH_2_O. The conidial titer was adjusted to 3×10^7^ conidia/ml. An aliquot of 45 μl of conidial suspension from both strains was mixed, and 80 μl of this final mixture were spread on VMM agar plates (60 x 15 mm). Plates were incubated for 4.5 h at 25°C or 3.5 h at 30°C. Agar squares of 1 cm^2^ were excised and observed with a Zeiss Axioskop 2 equipped with a Q Imaging Retiga-2000R camera (Surrey) using a 40x/1.30 Plan-Neofluar oil immersion objective and the iVision Mac4.5 software. Different strains were either discriminated by GFP or FM4-64 fluorescence, or FM4-64 fluorescence versus no fluorescence, if two wild isolates were analyzed. Communication frequencies were determined for at least 15 fields, depicting a total of at least 100 interactions with three biological replicates.

### Heterokaryon Assays

Conidia of strains bearing different auxotrophic markers (*his-3*, *ad-3B*, or *pyr-4*; S4 Table) were harvested as described above. The conidial titer of one strain was adjusted to 3 x 10^6^ conidia/ml, and the conidial titer of the forced communication partner (bearing a different auxotrophic marker) was adjusted to 3 x 10^5^ conidia/ml. A 150 μl spore suspension of both strains was mixed and spread on modified VMM agar that promotes colonial growth (FGSC, http://www.fgsc.net/Neurospora/NeurosporaProtocolGuide.htm). Due to the complementing auxotrophic markers, only heterokaryotic, prototrophic fusion products were able to grow on VMM. Plates were incubated at 30°C for 4 d, when cell-forming units/plate were documented.

### Confocal Microscopy

Cellular localization studies were performed with a Leica SD6000 microscope with a 100×1.4 NA oil-immersion objective equipped with a Yokogawa CSU-X1 spinning disk head, a 488 nm laser for GFP fluorescence, and a 563 nm laser for mCherry fluorescence controlled by the Metamorph software (Molecular Devices, Sunnyvale, CA). Conidia from strains expressing fluorophore-tagged proteins were prepared for microscopy as described above. For time-lapse studies, images were taken at 30 s intervals. The software ImageJ (http://imagej.nih.gov/ij/) was used for image processing.

For co-localization studies, heterokaryons were created by inoculating the center of a plate with a mixture of conidia of a strain expressing DOC-1-GFP and a strain expressing MAK-2-mCherry, or a strain expressing DOC-2-GFP and a strain expressing MAK-2-mCherry, respectively (S4 Table). Conidia bearing both GFP and mCherry fluorescent proteins were prepared and imaged as explained above.

### Bulked Segregant Analyses and Genome Resequencing

For DNA isolation, strains were grown on VMM agar plates covered with a disk of sterile cellophane at 30°C for 24 h. DNA was purified using the DNeasy Blood & Tissue kit (Qiagen Inc.). Equal amounts of DNA from 46 segregants (66 ng/segregant) were combined and used for library preparations using the TruSeq DNA LT Kit (Illumina). All paired end libraries were sequenced on a HiSeq2000 sequencing platform using standard Illumina operating procedures (Vincent J. Coates Genomics Sequencing Laboratory, Berkeley) to a read length of 100 nucleotides and a minimum mean depth of genome coverage of 71 for the sequenced libraries after filtering for low-quality reads, using the DepthofCoverage program from GATKv2.3–9 [92]. Low-quality reads were removed from the sequencing data using the Fastx toolkit (http://hannonlab.cshl.edu/fastx_toolkit/index.html). The filtered paired ends were regrouped by a custom perl script and mapped to the *N*. *crassa* genome FGSC 2489 v12 with the short read aligner Bowtie2.00 [93]. Read groups were added to sorted BAM files with Picard-tools v1.85 (http:broadinstitute.github.io/picard/) and SNP analysis performed with The Genome Analysis Tool Kit v2.3–9 after indel realignment with the RealignerTargetCreator and IndelRealigner programs from the GATK [92]. SNPs were confirmed by viewing the mapped polymorphisms on the Integrative Genomics Viewer v2.3 [94]. The mapped reads for the two parental strains (FGSC 2489 and JW258) plus the mapped reads for the 46 pooled segregants (FGSC 2489 communicators, CG1 or JW258 communicators, CG2) are available at the Sequence Read Archive (SRA) (http://www.ncbi.nlm.nih.gov/sra) (SRA311058).

### Sequence Analysis

The *doc-1* and *doc-2* sequences of *N*. *crassa* and *N*. *discreta* wild isolates were obtained by a BLAST search [95] using NCU07191 and NCU07192 from FGSC 2489 as a query against de novo sequence assemblies from 26 wild isolates [55]. For DNA sequence comparisons, the pairwise sequence alignment tool EMBOSS Needle from EMBL-EBI was used [96]. Codon alignments were carried out using Macse [97] and visualized and processed using JalView (http://www.jalview.org/). Modified multiple alignments were trimmed using Trimal [98]. Phylogenetic trees were inferred from trimmed alignments using the default pipeline from Phylogeny.fr (Muscle, Gblocks, Phyml [100 bootstraps])[99] and visualized using FigTree1.4 (http://tree.bio.ed.ac.uk/software/figtree/). To obtain DNA divergence statistics, the trimmed codon alignments of *doc-1*, *doc-2*, and *doc-3* sequences were sorted based on CGH groups. DnaSP5 was used to compute polymorphism and divergence and to test the standard neutral model using Tajima’s D [100]. Partitioned alignments for each locus were created using RAxML [101].

To detect recombination within CGHs in the region around *doc-1/2*, the program Rdp [67] was used, applying the “all methods mode” with default setting. Sequences of about 30 kbp surrounding *doc-1/2* were extracted from de novo genome sequence assemblies. Alignments were made using the program Mafft for each CGH group [102]; gaps were trimmed using Trimal [98].

## Supporting Information

S1 Alignment Fasta file of alignment in S2 Fig. (FASTA)

S2 Alignment Fasta file of alignment in S3 Fig. (FASTA)

S1 Data Excel spreadsheet containing, in separate sheets, the underlying numerical data and statistical analysis for Figs 1B, 3A–3C, S1A, S1B, S4A–S4E and S5D. (XLSX)

S1 Fig Communication of wild isolates with FGSC 2489. Self-communication and non-self-communication frequencies between germlings of different wild isolates and FGSC 2489. One-color bars denote self-communication frequencies between genetically identical germlings from a wild isolate, while two-color bars denote communication frequencies between a wild isolate and FGSC 2489. Experiments were performed in triplicates, with at least 100 germling pairs counted in each experiment. Black bars indicate standard deviation (Student's *t* test, *: *p* < 0.05, **: *p* < 0.01; see S1 Data for numerical values). (A) FGSC 2489 communicators (B) FGSC 2489 non-communicators.
(TIF)

S2 Fig Amino acid alignment of DOC-1, DOC-2 and DOC-3. The amino acid sequences of DOC-1, DOC-2, and DOC-3 from 26 *N*. *crassa* wild isolates and eight *N*. *discreta* wild isolates were used for the alignment. Alignments were carried out using Macse [97] and visualized and processed using JalView. Conserved amino acids are shaded. CGH1 isolates are shown in orange, CGH2 isolates are shown in light green, CGH3 isolates are shown in blue, CGH4 isolates are shown in purple, and CGH5 isolates are shown in dark green. The predicted OmpH-like domain of DOC-2 is highlighted in grey. (? = N in DNA sequence; see S1 Alignment for fasta file).
(TIF)

S3 Fig Nucleotide alignment of the genetic interval between NCU07188 and NCU07196 of CGH1 isolates. The DNA sequences of the genetic interval between NCU07188 and NCU07196 from six *N*. *crassa* wild isolates and FGSC 2489 (all CGH1) were used for the alignment. Conserved nucleotides are shaded. Note CGH1A- and CGH1B-specific indels between the isolates in the intergenic region between NCU07190 and *doc-1*, between *doc-1* and *doc-2*, and between *doc-2* and NCU07193. An ~8 kbp insertion downstream of NCU07193 was present in strain JW204 (see S2 Alignment for fasta file).
(JPG)

S4 Fig Germling communication frequencies of different *doc-1* and/or *doc-2* mutants. Conidia of the *doc-1* and/or *doc-2* mutants were mixed with conidia of wild isolates stained with FM4-64, and communication frequencies were assessed 4 h after inoculation. Graphs represent self-communication frequencies of *doc-1* and/or *doc-2* mutants (left bar), self-communication frequencies of a wild isolate (middle bar), and communication frequencies of the *doc-1* and/or *doc-2* germlings interacting with germlings from a wild isolate (right bar). (A) Complementation with *doc-1-gfp* restores communication phenotype of a Δ*doc-1* mutant. (B) Complementation with *doc-2-gfp* restores the communication phenotype of a *Δdoc-2* mutant. CG tester strains were FGSC 2489 (CG1), JW262 (CG2) and P4483 (CG3). (C) A mutant deleted for Δ*doc-1* and Δ*doc-2* displays robust chemotropic interactions with CG5 strains (JW220, JW242, JW75; top row). A reduction in communication was observed when Δ*doc-1* Δ*doc-2* germlings were paired with CGH2 (JW258), CGH3 (D113), or CGH4 (D111) germlings (bottom row). (D) The Δ*doc-1* Δ*doc-2* (*his-3*::*doc-1* ^*CG3*^ *doc-2* ^*CG3*^) germlings show reduced communication with CGH5 strains (JW242; left) but enhanced communication with the donor for *doc-1* ^*CG3*^ and *doc-2* ^*CG3*^ (P4471, CG3) (*Δdoc1Δdoc2* ^*CG3*^ = *Δdoc-1 Δdoc-2* [*his-3*::*doc-1* ^*CG3*^ *doc-2* ^*CG3*^]). (E) Quantitative results of experiments on forced communication (see Fig 3D) (CFU: Colony forming unit, Student's *t* test, **: *p* < 0.001; see S1 Data for numerical values).
(TIF)

S5 Fig Oscillation dynamics of DOC-1-GFP in hyphae and germlings. (A) Graphical representation of relative DOC-1-GFP fluorescence intensity (*y*-axis) at the tip of one homing hypha 1 (blue) and DOC-1-GFP fluorescence at the tip of its interaction partner (homing hyphae 2 [red]) when undergoing chemotropic interactions within a single colony over a 50 min time course (*x*-axis). S4 Movie served as basis for these measurements. (B) Graphical representation of relative DOC-1-GFP fluorescence intensity (*y*1-axis) at the CAT tip of one germling (green) and the relative SOFT-mCherry fluorescence intensity (*y*2-axis) at the CAT tip of its interaction partner germling (red) over a 50 min time course (*x*-axis). The oscillation interval was calculated to be 9 ± 1.24 min for both proteins. S3 Movie served as basis for the measurements. (C) Western blot of anti-GFP immunoprecipitated proteins probed with anti-GFP antibodies show that both fusion proteins are expressed (DOC-1-GFP and DOC-2-GFP ~120 kDa; GFP ~25 kDa). (D) SOFT-GFP oscillation intervals were measured in communicating FGSC 2489 germlings (CG1, *n* = 3) and in Δ*doc-1* Δ*doc-2* germlings (CG5, *n* = 4). There was no significant difference in oscillation timing detectable (*p* > 0.5; see S1 Data for numerical values).
(TIF)

S6 Fig Partitioned phylogenetic trees indicate congruent genealogies for *doc-1*, *doc-2*, and *doc-3*. *doc-1* (A), *doc-2* (B), and *doc-3* (C) sequences were divided into three regions (N-terminal, central, and C-terminal), and phylogenetic trees were built for each part. The tree structure for each region is similar to phylogenetic trees based on whole protein sequences (compare with Fig 7), suggesting that there is no recombination between the communication group haplotypes. Black bars indicate substitution rates. CGH1 isolates are shown in orange, CGH2 isolates are shown in light green, CGH3 isolates are shown in blue, CGH4 isolates are shown in purple, and CGH5 isolates are shown in dark green (see S2 Tree for Nexus file).
(TIF)

S7 Fig Detection of genetic recombination within isolates of the same CGH in the genetic interval between NCU07188 and NCU07195. *x*-axis shows the genomic position and *y*-axis shows log (p-val) for recombination events. Analyses were carried out using the program Rdp [67]. CGH3 isolates were not included in the analyses due to the small sample size. (CI = Confidence Interval).
(TIF)

S1 Movie Germlings from different CG1 strains, FGSC 2489 and P4472, show chemotropic interactions. Conidia of the strain FGSC 2489 expressing cytoplasmic GFP (green) were mixed with conidia of the strain P4472 stained with FM4-64 (red). Germination and communication was followed 4 h after inoculation. Pictures were taken every 30 s. Yellow arrows mark chemotropic interactions between genetically identical germlings. Red arrows mark chemotropic interactions between FGSC 2489 and P4472 (genetically different) germlings. Scale bar: 10 μm.
(AVI)

S2 Movie Germlings of strains from different communication groups (CG1, FGSC 2489 and CG2, JW161) avoid each other. Conidia of the strain FGSC 2489 expressing cytoplasmic GFP (green) were mixed with conidia of the strain JW161 stained with FM4-64 (red). Germination and communication was followed 4 h after inoculation. Pictures were taken every 30 s. Yellow arrows mark chemotropic interactions between genetically identical germlings. Black arrows mark genetically different germlings that do not show chemotropic interactions, although they are close enough to interact. Scale bar: 10 μm.
(AVI)

S3 Movie DOC-1-GFP and SOFT-mCherry oscillate with identical dynamics in opposing tips during chemotropic interactions. Time course of DOC-1-GFP and SOFT-mCherry localization to tips of interacting germlings. The oscillation of both proteins was observed. When SOFT-mCherry (red) localized to the tip of one germling, DOC-1-GFP (green) localized to the tip of the interaction partner. When SOFT-mCherry was absent from the tip of one germling, DOC-1-GFP was absent from the tip of the interaction partner.
(AVI)

S4 Movie DOC-1-GFP shows oscillatory localization to fusion points and punctae in hyphae during chemotropic interactions of fusion hyphae. Time course of DOC-1-GFP localization to interacting hyphae prior to cell fusion. DOC-1-GFP localized to the hyphal tip of a homing hyphae, followed by a disappearance and localization of DOC-1-GFP at the cell surface in the receptive hyphae. Pictures were taken every 30 s. Note that the formation and oscillation of DOC-1-GFP is restricted to the hyphal tip and last segment of the homing hyphae, which is separated by a septum.
(AVI)

S5 Movie Enforcement of oscillation of MAK-2-GFP in interacting germlings of different CGs. MAK-2-GFP in a *Δdoc-1 Δdoc-2* germling shows continued oscillation to the CAT tip if chemotropic interactions are established between *Δdoc-1 Δdoc-2* (CG5) and JW262 (CG2) germlings.
(AVI)

S1 Table Wild *Neurospora crassa* isolates used in this study, with communication group and CGH designation. (DOCX)

S2 Table Average number of nucleotide substitutions in alleles encoded by genes within the genetic interval NCU17048 to NCU07194 (Fig 2B) per site between each CGH pair. (DOCX)

S3 Table Tajima’s D statistics assessing selection acting on loci within the NCU17048 to NCU07194 genetic interval, including *doc-1*, *doc-2*, and *doc-3*. (DOCX)

S4 Table Laboratory and engineered *N*. *crassa* strains constructed for this study. (DOCX)

S5 Table Primers used in this study. (DOCX)

S1 Tree Nexus file of trees in Fig 7. (TXT)

S2 Tree Nexus file of trees in S6 Fig. (TXT)

## Abbreviations

CAT: conidial anastomosis tube
CG: communication group
CGH: communication group haplotype
GFP: green fluorescent protein
MAPK: mitogen-activated protein kinase
RFI: relative fluorescence intensity
SNP: single nucleotide polymorphism
