# Supplementary figures and images for: Characterization of Greenbeard Genes Involved in Long-Distance Kind Discrimination in a Microbial Eukaryote
Source: PLoS Biol. 2016 Apr 14;14(4):e1002431. doi: 10.1371/journal.pbio.1002431 (PMC4831770; doi:10.1371/journal.pbio.1002431)

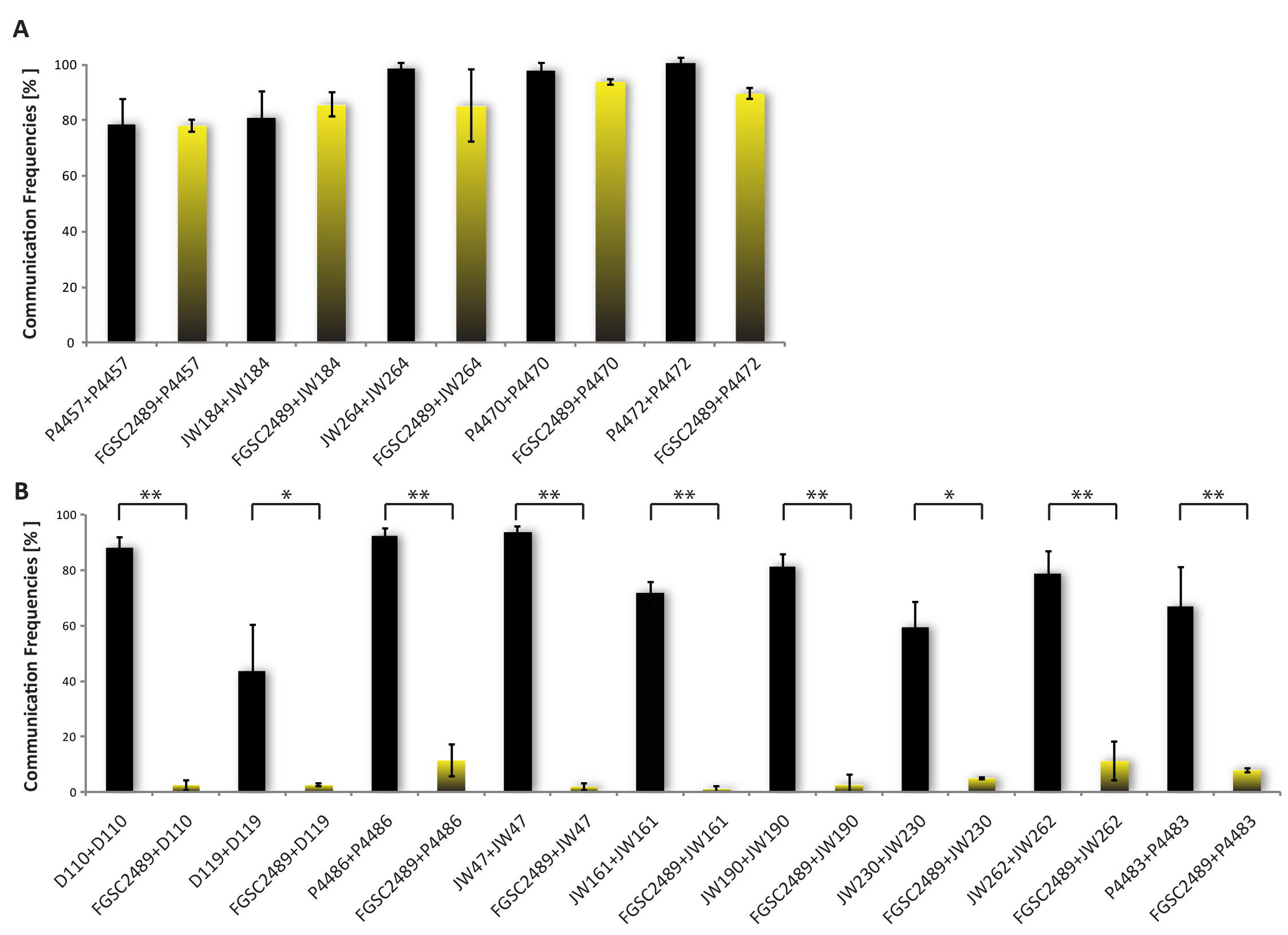

Supplement: S1 Fig — Self-communication and non-self-communication frequencies between germlings of different wild isolates and FGSC 2489. One-color bars denote self-communication frequencies between genetically identical germlings from a wild isolate, while two-color bars denote communication frequencies between a wild isolate and FGSC 2489. Experiments were performed in triplicates, with at least 100 germling pairs counted in each experiment. Black bars indicate standard deviation (Student's t test, *: p < 0.05, **: p < 0.01; see S1 Data for numerical values). (A) FGSC 2489 communicators (B) FGSC 2489 non-communicators. (TIF) [file pbio.1002431.s004.tif]

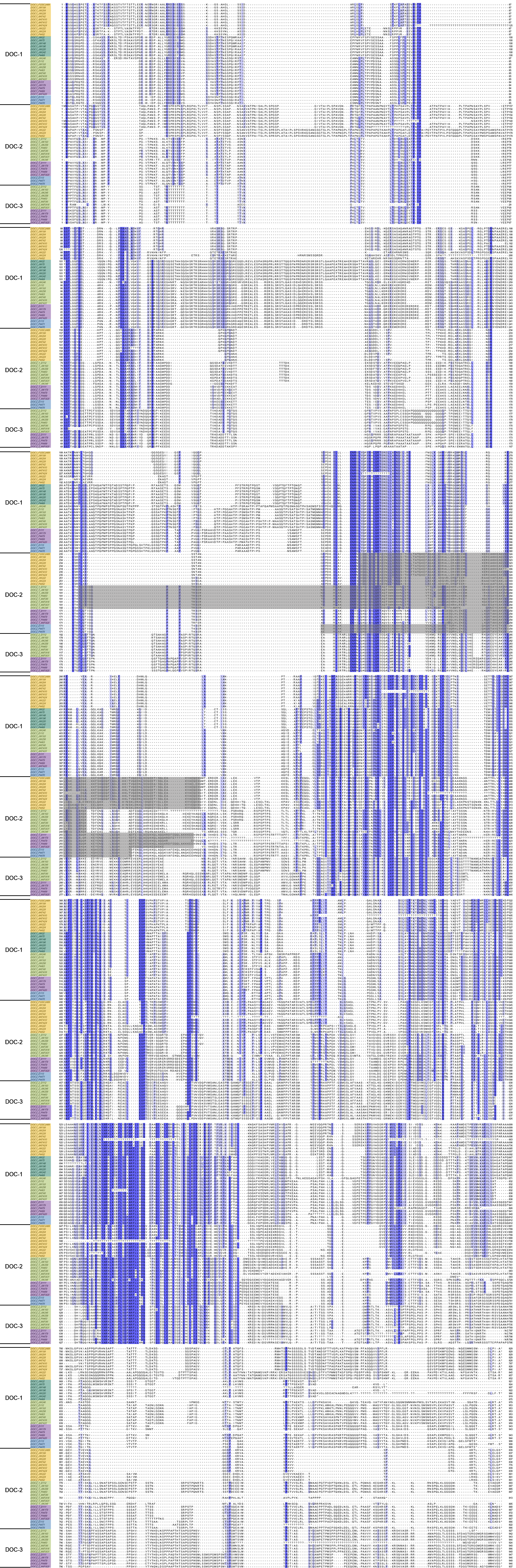

Supplement: S2 Fig — The amino acid sequences of DOC-1, DOC-2, and DOC-3 from 26 N. crassa wild isolates and eight N. discreta wild isolates were used for the alignment. Alignments were carried out using Macse [97] and visualized and processed using JalView. Conserved amino acids are shaded. CGH1 isolates are shown in orange, CGH2 isolates are shown in light green, CGH3 isolates are shown in blue, CGH4 isolates are shown in purple, and CGH5 isolates are shown in dark green. The predicted OmpH-like domain of DOC-2 is highlighted in grey. (? = N in DNA sequence; see S1 Alignment for fasta file). (TIF) [file pbio.1002431.s005.tif]

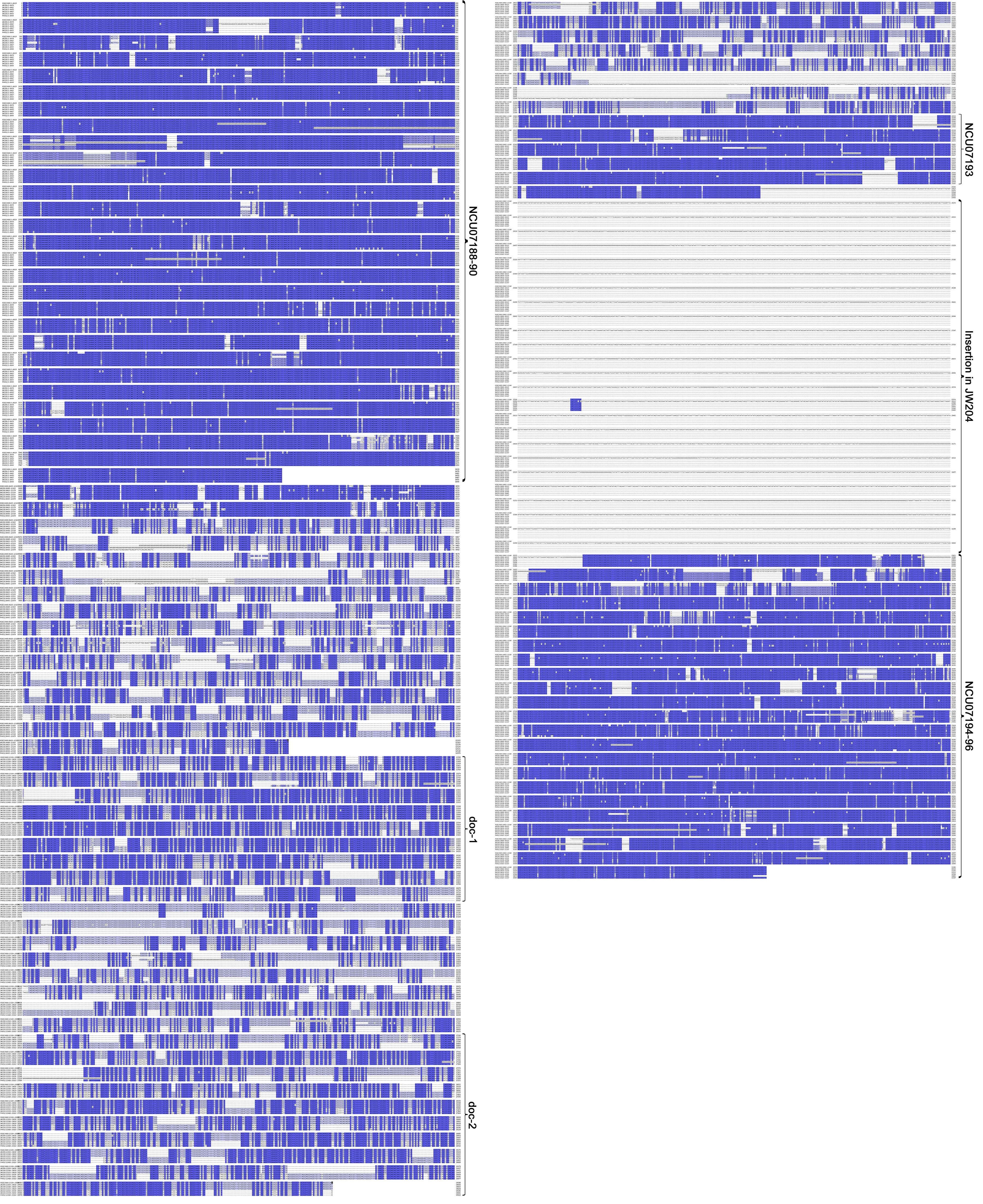

Supplement: S3 Fig — The DNA sequences of the genetic interval between NCU07188 and NCU07196 from six N. crassa wild isolates and FGSC 2489 (all CGH1) were used for the alignment. Conserved nucleotides are shaded. Note CGH1A- and CGH1B-specific indels between the isolates in the intergenic region between NCU07190 and doc-1, between doc-1 and doc-2, and between doc-2 and NCU07193. An ~8 kbp insertion downstream of NCU07193 was present in strain JW204 (see S2 Alignment for fasta file). (JPG) [file pbio.1002431.s006.jpg]

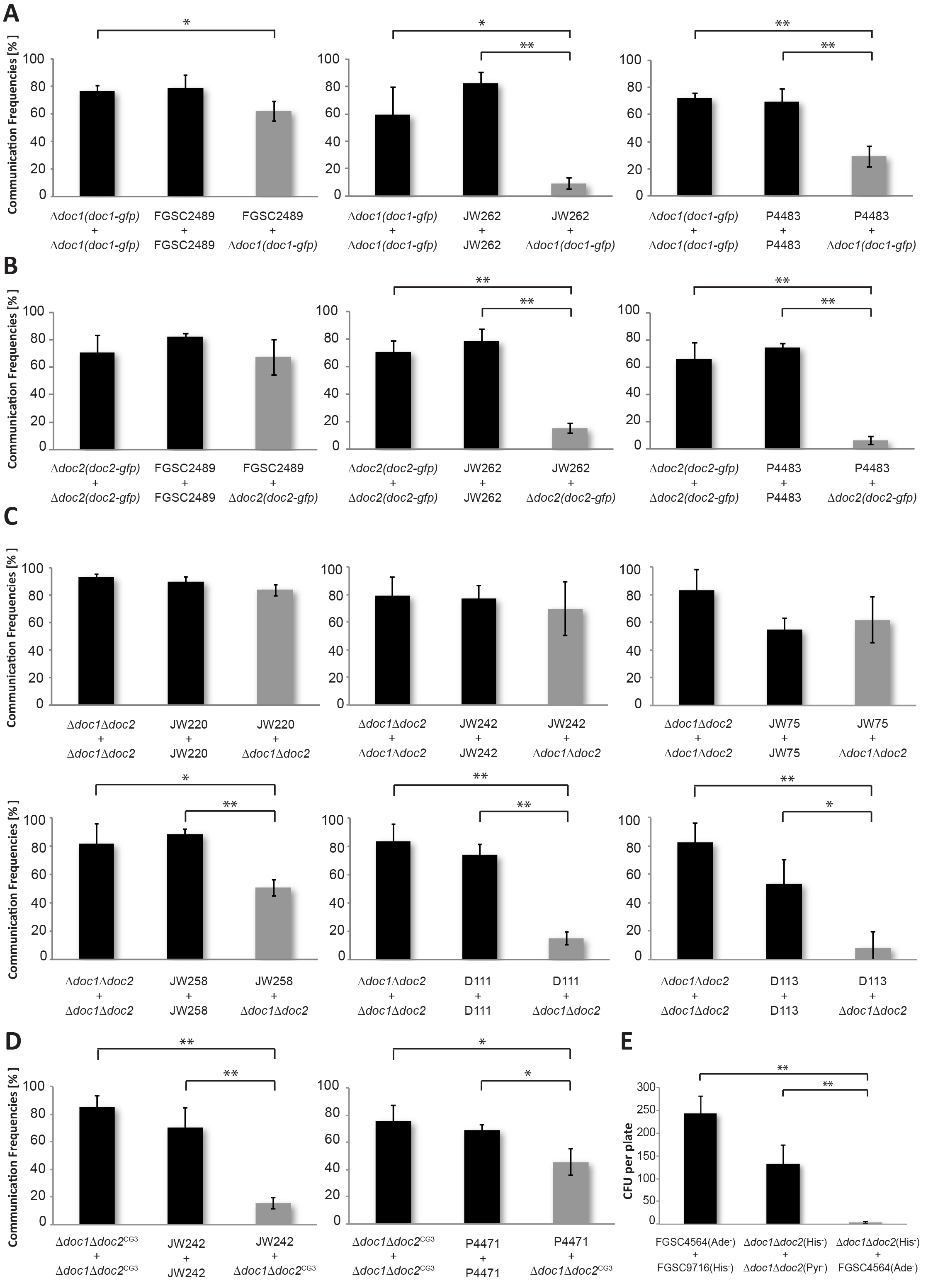

Supplement: S4 Fig — Conidia of the doc-1 and/or doc-2 mutants were mixed with conidia of wild isolates stained with FM4-64, and communication frequencies were assessed 4 h after inoculation. Graphs represent self-communication frequencies of doc-1 and/or doc-2 mutants (left bar), self-communication frequencies of a wild isolate (middle bar), and communication frequencies of the doc-1 and/or doc-2 germlings interacting with germlings from a wild isolate (right bar). (A) Complementation with doc-1-gfp restores communication phenotype of a Δdoc-1 mutant. (B) Complementation with doc-2-gfp restores the communication phenotype of a Δdoc-2 mutant. CG tester strains were FGSC 2489 (CG1), JW262 (CG2) and P4483 (CG3). (C) A mutant deleted for Δdoc-1 and Δdoc-2 displays robust chemotropic interactions with CG5 strains (JW220, JW242, JW75; top row). A reduction in communication was observed when Δdoc-1 Δdoc-2 germlings were paired with CGH2 (JW258), CGH3 (D113), or CGH4 (D111) germlings (bottom row). (D) The Δdoc-1 Δdoc-2 (his-3::doc-1 CG3 doc-2 CG3) germlings show reduced communication with CGH5 strains (JW242; left) but enhanced communication with the donor for doc-1 CG3 and doc-2 CG3 (P4471, CG3) (Δdoc1Δdoc2 CG3 = Δdoc-1 Δdoc-2 [his-3::doc-1 CG3 doc-2 CG3]). (E) Quantitative results of experiments on forced communication (see Fig 3D) (CFU: Colony forming unit, Student's t test, **: p < 0.001; see S1 Data for numerical values). (TIF) [file pbio.1002431.s007.tif]

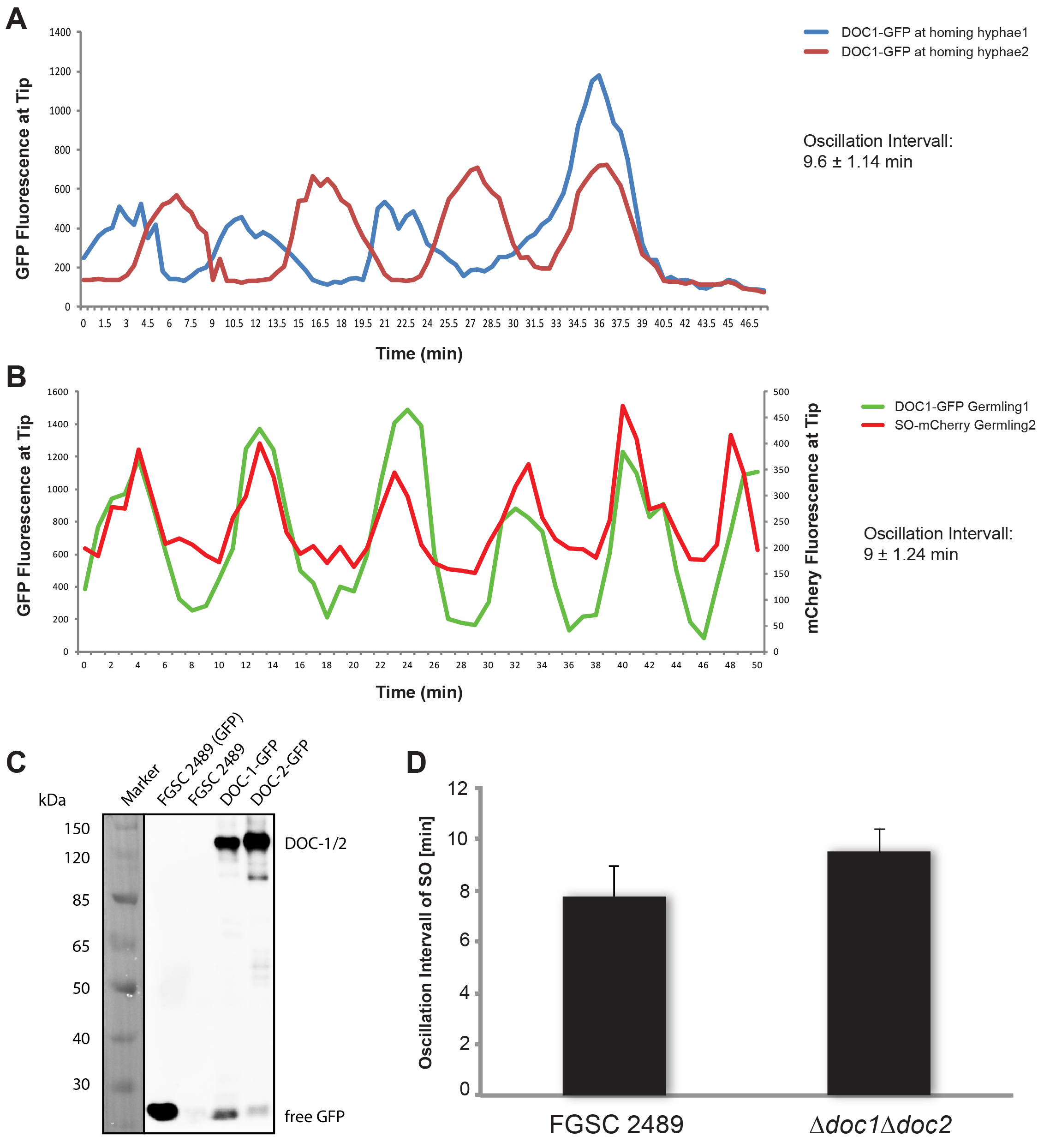

Supplement: S5 Fig — (A) Graphical representation of relative DOC-1-GFP fluorescence intensity (y-axis) at the tip of one homing hypha 1 (blue) and DOC-1-GFP fluorescence at the tip of its interaction partner (homing hyphae 2 [red]) when undergoing chemotropic interactions within a single colony over a 50 min time course (x-axis). S4 Movie served as basis for these measurements. (B) Graphical representation of relative DOC-1-GFP fluorescence intensity (y1-axis) at the CAT tip of one germling (green) and the relative SOFT-mCherry fluorescence intensity (y2-axis) at the CAT tip of its interaction partner germling (red) over a 50 min time course (x-axis). The oscillation interval was calculated to be 9 ± 1.24 min for both proteins. S3 Movie served as basis for the measurements. (C) Western blot of anti-GFP immunoprecipitated proteins probed with anti-GFP antibodies show that both fusion proteins are expressed (DOC-1-GFP and DOC-2-GFP ~120 kDa; GFP ~25 kDa). (D) SOFT-GFP oscillation intervals were measured in communicating FGSC 2489 germlings (CG1, n = 3) and in Δdoc-1 Δdoc-2 germlings (CG5, n = 4). There was no significant difference in oscillation timing detectable (p > 0.5; see S1 Data for numerical values). (TIF) [file pbio.1002431.s008.tif]

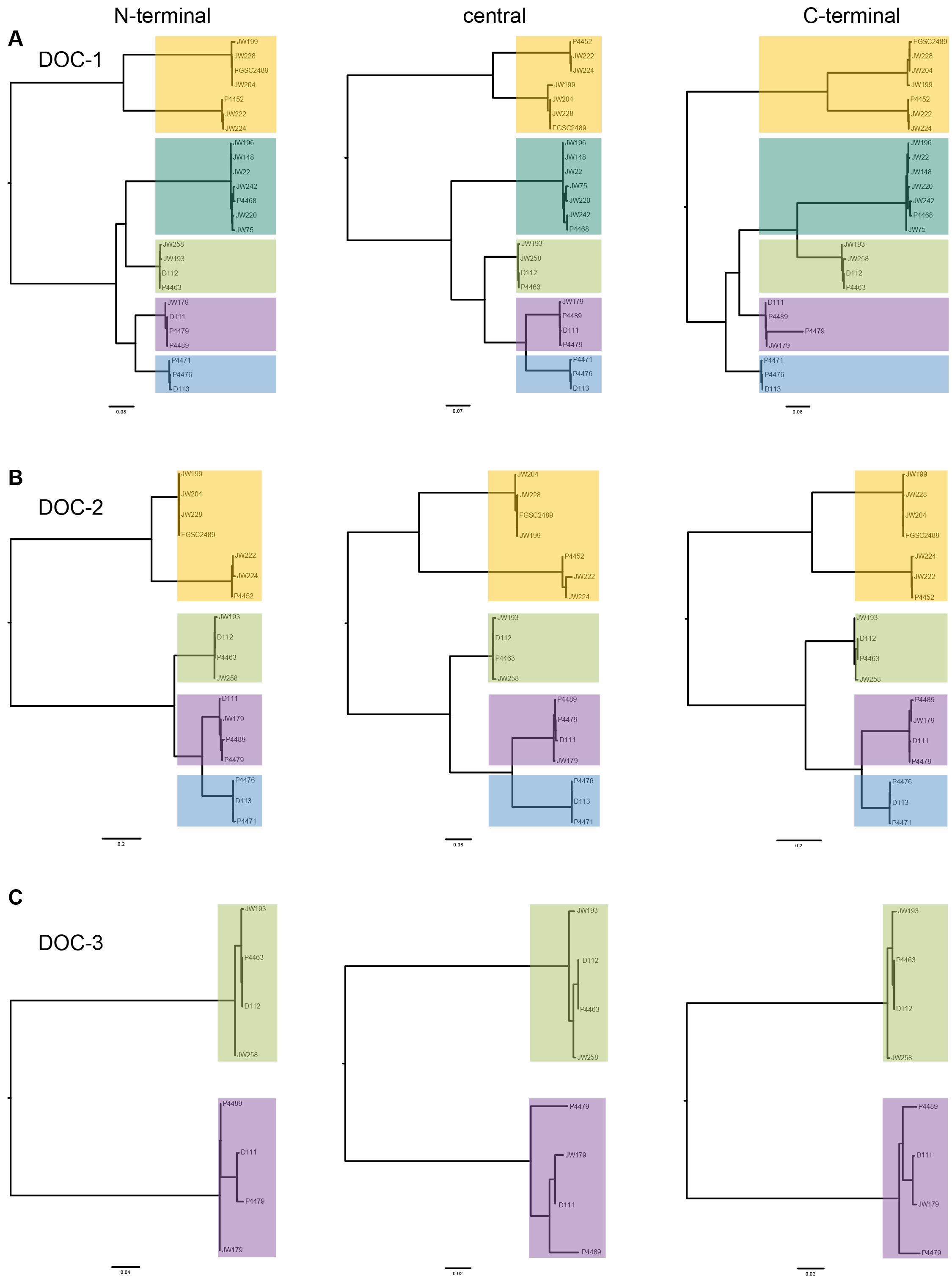

Supplement: S6 Fig — doc-1 (A), doc-2 (B), and doc-3 (C) sequences were divided into three regions (N-terminal, central, and C-terminal), and phylogenetic trees were built for each part. The tree structure for each region is similar to phylogenetic trees based on whole protein sequences (compare with Fig 7), suggesting that there is no recombination between the communication group haplotypes. Black bars indicate substitution rates. CGH1 isolates are shown in orange, CGH2 isolates are shown in light green, CGH3 isolates are shown in blue, CGH4 isolates are shown in purple, and CGH5 isolates are shown in dark green (see S2 Tree for Nexus file). (TIF) [file pbio.1002431.s009.tif]

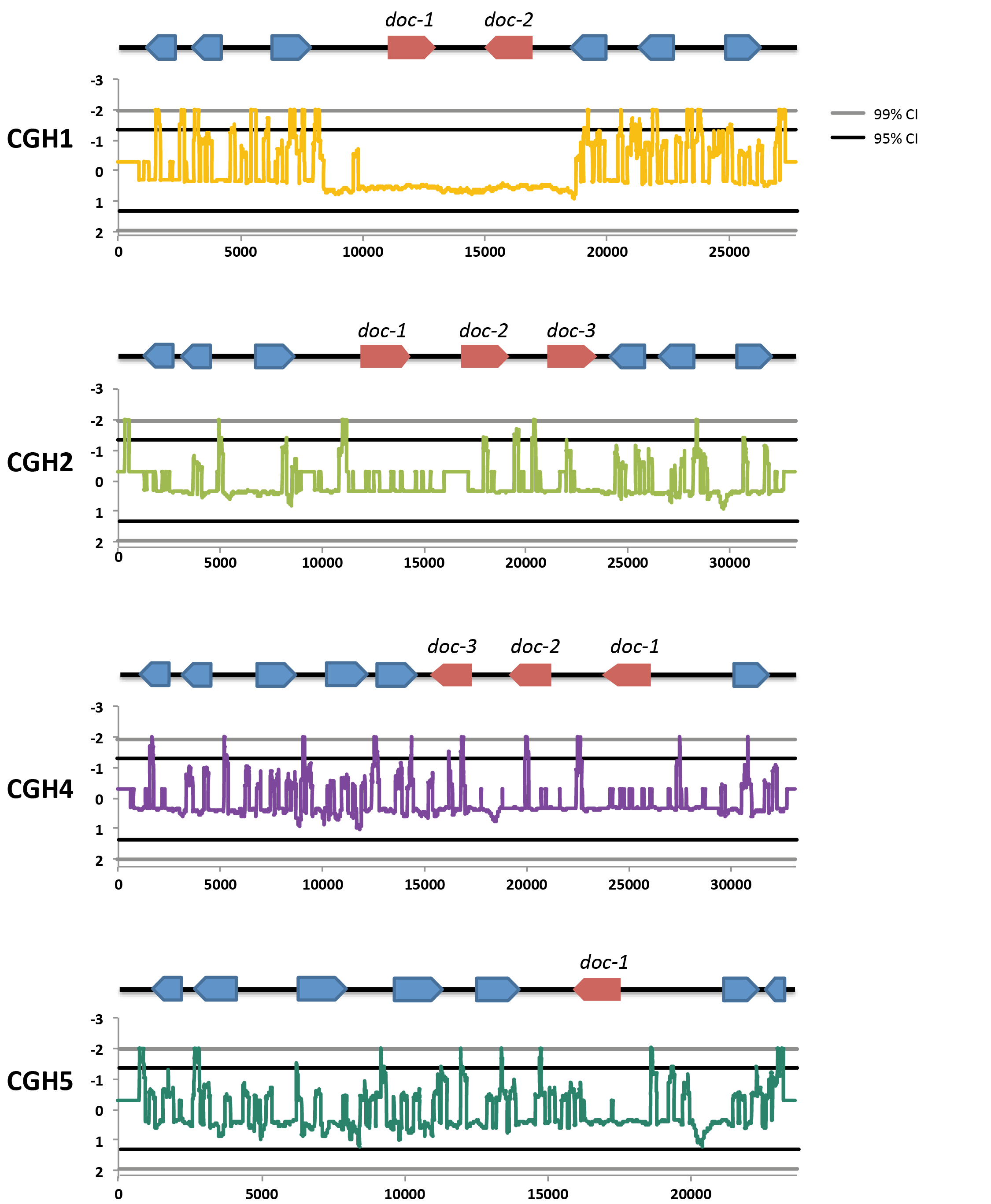

Supplement: S7 Fig — x-axis shows the genomic position and y-axis shows log (p-val) for recombination events. Analyses were carried out using the program Rdp [67]. CGH3 isolates were not included in the analyses due to the small sample size. (CI = Confidence Interval). (TIF) [file pbio.1002431.s010.tif]
