# Supplementary material for: Characterization of Greenbeard Genes Involved in Long-Distance Kind Discrimination in a Microbial Eukaryote
Source: PLoS Biol. 2016 Apr 14;14(4):e1002431. doi: 10.1371/journal.pbio.1002431 (PMC4831770; doi:10.1371/journal.pbio.1002431)
Supplement: S1 Table — (DOCX) [file pbio.1002431.s016.docx]

| **Strain** | **Reference ^[1]^** | **Collection Site** | **mating type** | **Communication group** | **CGH organization** |
| --- | --- | --- | --- | --- | --- |
| **P4452** | [1] | Franklin, Louisiana | a | 1 | 1 |
| **P4463** | [1] | Franklin, Louisiana | a | 2 | 2 |
| **P4468** | [2] | Franklin, Louisiana | A | 2 | 5 |
| **P4471** | [1] | Franklin, Louisiana | a | 3 | 3 |
| **P4476** | [1] | Franklin, Louisiana | a | 3 | 3 |
| **P4479** | [1] | Franklin, Louisiana | a | 2/3 | 4 |
| **P4489** | [1] | Franklin, Louisiana | a | 2/3 | 4 |
| **D111** | [3] | Franklin, Louisiana | a | 2/3 | 4 |
| **D112** | [3] | Franklin, Louisiana | A | 2 | 2 |
| **D113** | [3] | Franklin, Louisiana | a | 3 | 3 |
| **JW22** | [4] | Elizabeth, Louisiana | A | 2 | 5 |
| **JW75** | [4] | Houma, Louisiana | a | 2 | 5 |
| **JW148** | [4] | NorthsidePlantation.Louisiana | A | 2 | 5 |
| **JW179** | [4] | Roanoke, Louisiana | a | 2/3 | 4 |
| **JW193** | [4] | Elizabeth, Louisiana | a | 2 | 2 |
| **JW196** | [4] | NorthsidePlantation,Louisiana | A | 2 | 5 |
| **JW199** | [4] | Franklin. Louisiana | A | 1 | 1 |
| **JW204** | [4] | Bayou Chicot Louisiana | A | 1 | 1 |
| **JW220** | [4] | Roanoke, Louisiana | A | 2 | 5 |
| **JW222** | [4] | Coon, Louisiana | a | 1 | 1 |
| **JW224** | [4] | Coon, Louisiana | a | 1 | 1 |
| **JW228** | [4] | Georgia Plantation, Louisiana | a | 1 | 1 |
| **JW242** | [4] | Roanoke, Louisiana | A | 2 | 5 |
| **JW246** | [4] | Welsh, Louisiana | a | n.d. | 2 |
| **JW258** | [4] | Iowa, Louisiana | a | 2 | 2 |
| 847 | [5] | Louisiana | A | 1 | n.d |
| 1693 | [5] | Louisiana | a | 1 | n.d |
| P4448 | [6] | Franklin, Louisiana | A | 2 | n.d |
| P4449 | [6] | Franklin, Louisiana | a | 3 | n.d |
| P4450 | [1] | Franklin, Louisiana | a | 2 | n.d |
| P4451 | [1] | Franklin, Louisiana | a | 2 | n.d |
| P4453 | [6] | Franklin, Louisiana | A | 2 | n.d |
| P4455 | [1] | Franklin, Louisiana | a | 3 | n.d |
| P4457 | [1] | Franklin, Louisiana | a | 1 | n.d |
| P4459 | [1] | Franklin, Louisiana | a | 2 | n.d |
| P4465 | [1] | Franklin, Louisiana | a | 2 | n.d |
| P4469 | [1] | Franklin, Louisiana | a | 3 | n.d |
| P4470 | [1] | Franklin, Louisiana | A | 1 | n.d |
| P4472 | [1] | Franklin, Louisiana | a | 1 | n.d |
| P4483 | [1] | Franklin, Louisiana | a | 3 | n.d |
| P4486 | [1] | Franklin, Louisiana |  | 3 | n.d |
| P4487 | [1] | Franklin, Louisiana |  | 2 | n.d |
| P4494 | [1] | Franklin, Louisiana | a | 3 | n.d |
| P4496 | [1] | Franklin, Louisiana | a | 3 | n.d |
| P4497 | [1] | Franklin, Louisiana | a | 2 | n.d |
| P4498 | [1] | Franklin, Louisiana | a | 3 | n.d |
| P4500 | [6] | Franklin, Louisiana | a | 2 | n.d |
| D110 | [6] | Franklin, Louisiana | A | 2 | n.d |
| D114 | [6] | Franklin, Louisiana | A | n.d. | n.d |
| D116 | [6] | Franklin, Louisiana | a | 3 | n.d |
| D117 | [6] | Franklin, Louisiana | a | 2 | n.d |
| D118 | [6] | Franklin, Louisiana | a | 2 | n.d |
| D119 | [6] | Franklin, Louisiana | a | 2 | n.d |
| D143 | [6] | Marrero, Louisiana | A | 1 | n.d |
| JW9 | [4] | Welsh, Louisiana | A | 1 | n.d |
| JW10 | [4] | Welsh, Louisiana | A | 1 | n.d |
| JW16 | [4] | Welsh, Louisiana | a | 1 | n.d |
| JW20 | [4] | Ravenswood,  Louisiana | A | 1 | n.d |
| JW24 | [4] | Elizabeth, Louisiana | A | 2 | n.d |
| JW27 | [4] | Bayou Chicot, Louisiana | A | 1 | n.d |
| JW59 | [4] | Coon, Louisiana | a | 2 | n.d |
| JW60 | [4] | Coon, Louisiana | a | 1 | n.d |
| JW66 | [4] | Sugartown, Louisiana | a | 2 | n.d |
| JW70 | [4] | Coon, Louisiana | A | 3 | n.d |
| JW76 | [4] | Houma, Louisiana | a | 2 | n.d |
| JW96 | [4] | Roanoke, Louisiana | A | 1 | n.d |
| JW156 | [4] | Houma, Louisiana |  | 2 | n.d |
| JW159 | [4] | Houma, Louisiana | a | 1 | n.d |
| JW160 | [4] | Iowa, Louisiana | A | 1 | n.d |
| JW161 | [4] | Iowa, Louisiana | A | 2 | n.d |
| JW162 | [4] | Iowa, Louisiana | a | 3 | n.d |
| JW164 | [4] | Marrero, Louisiana | a | 1 | n.d |
| JW167 | [4] | Roanoke, Louisiana | a | n.d. | n.d |
| JW169 | [4] | Houma, Louisiana | a | 2 | n.d |
| JW171 | [4] | Houma, Louisiana | a | 2 | n.d |
| JW172 | [4] | Houma, Louisiana | A | 3 | n.d |
| JW174 | [4] | Houma, Louisiana | a | n.d. | n.d |
| JW176 | [4] | Welsh, Louisiana | a | 3 | n.d |
| JW178 | [4] | Roanoke, Louisiana | a | 3 | n.d |
| JW180 | [4] | Roanoke, Louisiana | a | n.d. | n.d |
| JW182 | [4] | Iowa, Louisiana | a | 1 | n.d |
| JW184 | [4] | Iowa, Louisiana | a | 1 | n.d |
| JW187 | [4] | Elizabeth, Louisiana | A | 1 | n.d |
| JW188 | [4] | Elizabeth, Louisiana | A | 2 | n.d |
| JW190 | [4] | Elizabeth, Louisiana | A | 2 | n.d |
| JW200 | [4] | Houma, Louisiana | a | 2 | n.d |
| JW202 | [4] | Elizabeth, Louisiana | a | n.d. | n.d |
| JW206 | [4] | Coon, Louisiana | A | n.d. | n.d |
| JW209 | [4] | Fred, Louisiana | a | 2 | n.d |
| JW210 | [4] | Franklin, Louisiana | a | 3 | n.d |
| JW216 | [4] | Welsh, Louisiana | a | 1 | n.d |
| JW218 | [4] | Welsh, Louisiana | a | 2 | n.d |
| JW230 | [4] | Georgia Plantation, Louisiana | a | 3 | n.d |
| JW233 | [4] | Houma, Louisiana | a | 2 | n.d |
| JW234 | [4] | Houma, Louisiana | A | 2 | n.d |
| JW238 | [4] | Welsh, Louisiana | A | 1 | n.d |
| JW240 | [4] | Roanoke, Louisiana | A | n.d. | n.d |
| JW245 | [4] | Roanoke, Louisiana | a | 2 | n.d |
| JW248 | [4] | Roanoke, Louisiana | A | 2 | n.d |
| JW250 | [4] | Sugartown, Louisiana | A | 2 | n.d |
| JW252 | [4] | Sugartown, Louisiana | A | 2 | n.d |
| JW254 | [4] | Iowa, Louisiana | a | 2 | n.d |
| JW256 | [4] | Iowa, Louisiana | a | n.d. | n.d |
| JW260 | [4] | Elizabeth, Louisiana | A | 2 | n.d |
| JW261 | [4] | Elizabeth, Louisiana | A | 2 | n.d |
| JW262 | [4] | Elizabeth, Louisiana | a | 2 | n.d |
| JW266 | [4] | Elizabeth, Louisiana | A | 2 | n.d |
| JW1 |  | Costa Rica | n.d | 2 | n.d |
| JW3 |  | Panama | n.d | 2 | n.d |
| JW5 | [4] | Panama | n.d | 2 | n.d |
| JW7 | [4] | Panama | n.d | 1 | n.d |
| JW11 |  | Puerto Rico | n.d | 1 | n.d |
| JW14 |  | Costa Rica | n.d | 2 | n.d |
| JW15 |  | Panama | n.d | 1 | n.d |
| JW35 |  | Florida | n.d | 3 | n.d |
| JW36 |  | Haiti | n.d | 1 | n.d |
| JW39 |  | Haiti | n.d | 1 | n.d |
| JW42 |  | Haiti | n.d | 3 | n.d |
| JW43 |  | Haiti | n.d | 2 | n.d |
| JW45 |  | Haiti | n.d | 2 | n.d |
| JW46 |  | Haiti | n.d | 2 | n.d |
| JW47 |  | Haiti | n.d | 2 | n.d |
| JW49 |  | Haiti | n.d | 2 | n.d |
| JW50 |  | Haiti | n.d | 2 | n.d |
| JW52 |  | Venezuela | n.d | 1 | n.d |
| JW54 |  | Haiti | n.d | 1 | n.d |
| JW56 |  | Digitima Creek, Guiana | n.d | 1 | n.d |
| JW57 |  | Torani Canal, Guyana | n.d | 1 | n.d |
| JW64 |  | Spurger, Texas | n.d | 2 | n.d |
| JW65 |  | Spurger, Texas | n.d | 2 | n.d |

**^1^References**

1. Bhat A, Noubissi FK, Vyas M, Kasbekar DP (2003) Genetic analysis of wild-isolated *Neurospora crassa* strains identified as dominant suppressors of repeat-induced point mutation. Genetics 164: 947-61. pmid: 12871906.

2. Smith ML, Micali OC, Hubbard SP, Mir-Rashed N, Jacobson DJ, Glass NL (2000) Vegetative incompatibility in the *het-6* region of *Neurospora crassa* is mediated by two linked genes. Genetics 155: 1095-104. pmid: 10880472.

3. Dettman JR, Jacobson DJ, Taylor JW (2003) A multilocus genealogical approach to phylogenetic species recognition in the model eukaryote *Neurospora*. Evol 7: 2703-20.

4. Palma-Guerrero J, Hall CR, Kowbel D, Welch J, Taylor JW, Brem RB, et al (2013) Genome wide association identifies novel loci involved in fungal communication. PLoS Genet 9: e1003669. doi: 10.1371/journal.pgen.1003669. pmid: 23935534.

5. Wu J, Saupe SJ, Glass NL (1998) Evidence for balancing selection operating at the *het-c* heterokaryon incompatibility locus in a group of filamentous fungi. Proc Natl Acad Sci U S A 95: 12398-403. pmid: 9770498.

6. Dettman JR, Jacobson DJ, Taylor JW (2006) Multilocus sequence data reveal extensive phylogenetic species diversity within the *Neurospora discreta* complex. Mycologia 98: 436-46. pmid: 17040072.
