# Supplementary material for: Characterization of Greenbeard Genes Involved in Long-Distance Kind Discrimination in a Microbial Eukaryote
Source: PLoS Biol. 2016 Apr 14;14(4):e1002431. doi: 10.1371/journal.pbio.1002431 (PMC4831770; doi:10.1371/journal.pbio.1002431)
Supplement: S2 Table — (DOCX) [file pbio.1002431.s017.docx]

| **NCU17048** | | | | | | |
| --- | --- | --- | --- | --- | --- | --- |
|  | **CGH1** | **CGH2** | **CGH3** | **CGH4** | **CGH5** |  |
| **CGH1** | 0.02 | 0.02 | 0.03 | 0.03 | 0.03 |  |
| **CGH2** |  | 0.02 | 0.03 | 0.03 | 0.03 |  |
| **CGH3** |  |  | 0.02 | 0.03 | 0.03 |  |
| **CGH4** |  |  |  | 0.02 | 0.03 |  |
| **CGH5** |  |  |  |  | 0.02 |  |
| **NCU07190** | | | | | |  |
|  | **CGH1** | **CGH2** | **CGH3** | **CGH4** | **CGH5** |  |
| **CGH1** | 0.01 | 0.02 | 0.02 | 0.02 | 0.02 |  |
| **CGH2** |  | 0.01 | 0.02 | 0.02 | 0.02 |  |
| **CGH3** |  |  | 0.01 | 0.02 | 0.02 |  |
| **CGH4** |  |  |  | 0.02 | 0.02 |  |
| **CGH5** |  |  |  |  | 0.02 |  |
| ***doc-1*** | | | | | |  |
|  | **CGH1** | **CGH2** | **CGH3** | **CGH4** | **CGH5** |  |
| **CGH1** | 0.18 | 0.66 | 0.65 | 0.68 | 0.74 |  |
| **CGH2** |  | 0.00 | 0.31 | 0.3 | 0.39 |  |
| **CGH3** |  |  | 0.00 | 0.22 | 0.46 |  |
| **CGH4** |  |  |  | 0.02 | 0.46 |  |
| **CGH5** |  |  |  |  | 0.01 |  |
| ***doc-2*** | | | | |  |  |
|  | **CGH1** | **CGH2** | **CGH3** | **CGH4** |  |  |
| **CGH1** | 0.21 | 0.85 | 0.87 | 0.84 |  |  |
| **CGH2** |  | 0.01 | 0.41 | 0.41 |  |  |
| **CGH3** |  |  | 0.00 | 0.26 |  |  |
| **CGH4** |  |  |  | 0.01 |  |  |
| ***doc-3*** | | | | |  |  |
|  | **CGH2** | **CGH4** |  |  |  |  |
| **CGH2** | 0.04 | 0.27 |  |  |  |  |
| **CGH4** |  | 0.00 |  |  |  |  |
| **NCU07193** | | | | | |  |
|  | **CGH1** | **CGH2** | **CGH3** | **CGH4** | **CGH5** |  |
| **CGH1** | 0.02 | 0.03 | 0.02 | 0.03 | 0.04 |  |
| **CGH2** |  | 0.02 | 0.03 | 0.03 | 0.03 |  |
| **CGH3** |  |  | 0.02 | 0.03 | 0.04 |  |
| **CGH4** |  |  |  | 0.02 | 0.03 |  |
| **CGH5** |  |  |  |  | 0.02 |  |
| **NCU07194** | | | | | |  |
|  | **CGH1** | **CGH2** | **CGH3** | **CGH4** | **CGH5** |  |
| **CGH1** | 0.03 | 0.05 | 0.04 | 0.06 | 0.06 |  |
| **CGH2** |  | 0.03 | 0.05 | 0.05 | 0.04 |  |
| **CGH3** |  |  | 0.02 | 0.05 | 0.05 |  |
| **CGH4** |  |  |  | 0.04 | 0.05 |  |
| **CGH5** |  |  |  |  | 0.03 |  |

^1^Nucleotide divergence was measured by K (JC-Total) using DnaSP5 with Jukes and Cantor correction [1].

1. Librado P, Rozas J (2009) DnaSP v5: a software for comprehensive analysis of DNA polymorphism data. Bioinformatics 25: 1451-2. doi: 10.1093/bioinformatics/btp187. pmid: 19346325.
