# Supplementary material for: Characterization of Greenbeard Genes Involved in Long-Distance Kind Discrimination in a Microbial Eukaryote
Source: PLoS Biol. 2016 Apr 14;14(4):e1002431. doi: 10.1371/journal.pbio.1002431 (PMC4831770; doi:10.1371/journal.pbio.1002431)
Supplement: S3 Table — (DOCX) [file pbio.1002431.s018.docx]

| **Locus** | **Tajima’s D ^[1]^** | **p-value** |
| --- | --- | --- |
| ***doc-1* (all)** | 2.69932 | p<0.01 |
| **CGH1** | 2.26796 | p<0.01 |
| **CGH2** | -0.07075 | p>0.1 |
| **CGH3** | na | na |
| **CGH4** | 0.82282 | p>0.1 |
| **CGH5** | 0.001310 | p>0.1 |
| ***doc-2* (all)** | 2.65686 | p<0.01 |
| **CGH1** | 2.27628 | p<0.01 |
| **CGH2** | 0.33502 | p>0.1 |
| **CGH3** | na | na |
| **CGH4** | 0.71703 | p>0.1 |
| ***doc-3* (all)** | 2.20533 | p<0.05 |
| **CGH2** | 0.07271 | p>0.1 |
| **CGH4** | -0.27601 | p>0.1 |
| **NCU07190** | 0.53937 | p>0.1 |
| **NCU07193** | 0.16897 | p>0.1 |
| **NCU07194** | 0.88334 | p>0.1 |
| **NCU17048** | 0.36136 | p>0.1 |

^1.^ Tajima F (1989) Statistical method for testing the neutral mutation hypothesis by DNA polymorphism. Genetics 123: 585-95. pmid: 2513255.
