# Supplementary material for: Characterization of Greenbeard Genes Involved in Long-Distance Kind Discrimination in a Microbial Eukaryote
Source: PLoS Biol. 2016 Apr 14;14(4):e1002431. doi: 10.1371/journal.pbio.1002431 (PMC4831770; doi:10.1371/journal.pbio.1002431)
Supplement: S4 Table — (DOCX) [file pbio.1002431.s019.docx]

| **Name** | **Genotype** | **Reference** |
| --- | --- | --- |
| FGSC2489 | Oak Ridge WT *A* | FGSC |
| FGSC4200 | *a* | FGSC |
| FGSC6103 | *his-3 A* | FGSC |
| FGSC9716 | *his-3 a* | FGSC |
| FGSC9718 | *∆mus-51::bar^+^ a* | FGSC |
| FGSC2489-gfp | *his-3::Pccg1-gfp A* | [1] |
|  | *his-3::Ptef1-mak-2-mCherry* | [1] |
|  | *his-3::Ptef1-so-mCherry* | [1] |
|  | *his-3::Ptef1-ham-5-gfp A* | [1] |
| ∆doc-1 | *∆doc-1::hyg^R^ a* | this study |
| ∆doc-1 his^-^ | *his-3; ∆doc-1::hyg^R^ A* | this study |
| ∆doc-1-gfp | *his-3::Pccg1-gfp; ∆doc-1:hyg^R^ A* | this study |
| JH1 | *his-3::Pccg1-doc-1-gfp A* | this study |
| JH2 | *his-3::Ptef1-doc-1-gfp A* | this study |
| JH3 | *his-3::doc1-gfp; ∆doc-1::hyg^R^ A* | this study |
| JH4 | *his-3::Pccg1-doc1-gfp; ∆doc-1::hyg^R^ A* | this study |
| JH6 | *his-3::Ptef1-gfp-doc1; ∆doc-1::hyg^R^ A* | this study |
| ∆doc-2 | *∆doc-2::hyg^R^ a* | this study |
| ∆doc-2 his^-^ | *his-3; ∆doc-2::hyg^R^ A* | this study |
| ∆doc-2-gfp | *his-3::Pccg1-gfp; ∆doc-1::hyg^R^ A* | this study |
| JH7 | *his-3::Pccg1-doc-2-gfp A* | this study |
| JH8 | *his-3::Pdoc2-doc2-gfp; ∆doc-2::hyg^R^ A* | this study |
| JH9 | *his-3::Pccg1-doc2-gfp; ∆doc-2::hyg^R^ A* | this study |
| JH11 | *his-3::Ptef1-gfp-doc-2; ∆doc-2::hyg^R^ A* | this study |
| JH12 | *his-3::Pccg1-gfp; ∆doc-1-∆doc-2::hyg^R^ A* | this study |
| JH13 | *his-3; ∆doc-1-∆doc-2::hyg^R^ a* | this study |
| JH14 | *pyr-4; ∆doc-1-∆doc-2::hyg^R^ a* | this study |
| JH15 | *his-3::doc-1^CG3^ doc-2^CG3^; ∆doc-1-∆doc-2::hyg^R^* | this study |

1. Jonkers W, Leeder AC, Ansong C, Wang Y, Yang F, Starr TL, et al (2014) HAM-5 functions as a MAP kinase scaffold during cell fusion in *Neurospora crassa*. PLoS Genet 10: e1004783. doi: 10.1371/journal.pgen.1004783. pmid: 25412208.
