# Supplementary material for: Characterization of Greenbeard Genes Involved in Long-Distance Kind Discrimination in a Microbial Eukaryote
Source: PLoS Biol. 2016 Apr 14;14(4):e1002431. doi: 10.1371/journal.pbio.1002431 (PMC4831770; doi:10.1371/journal.pbio.1002431)
Supplement: S5 Table — (DOCX) [file pbio.1002431.s020.docx]

| **Name** | **Sequence (5'-3')** |
| --- | --- |
| NCU07191 5' integration | CCACTACACTAGCCCGCTGAC |
| NCU07191_gen_f | CATCAACCGTGACCTTCACATC |
| NCU07191_gen_r | CTCCTTGAGCTTCACAAACTCC |
| NCU07191_start_XbaI | TCTAGAATGAGTAGCGGCAAGGGCTCTCC |
| NCU07191_end_PacI | TTAATTAAAGCAATAGGCAAATCCATACTTCCCATC |
| NCU07192 5' integration | CTTGTCCTCGGGCTAACATGC |
| NCU07192_gen_f | CGTTCTCTACGCCATGCTCAC |
| NCU07192_gen_r | CCAACCATACCCGTGCCTTAC |
| NCU07192_start_XbaI | TCTAGAATGATGGCCTCTGCAACCCCC |
| NCU07192_end_PacI | TTAATTAATGAACCCAGCTCAAGAGTCAACGTTC |
| 07191_promoter_f_NotI | GCGGCCGCGGACTACGGTAGGAACATGG |
| 07191_promoter_r_XbaI | TCTAGATGTTCCTCCTTTCACAAAGGTTGTG |
| NCU07191 5' flank f-primer | GTAACGCCAGGGTTTTCCCAGTCACGACGGGACTACGGTAGGAACATGG |
| NCU07191_5'_fusion_r | GCTCCTTCAATATCATCTTCTGTCTCCGACTACAGCGCGTCAACTTCC |
| NCU07192 5' flank f-primer | GTAACGCCAGGGTTTTCCCAGTCACGACGCTCCAGATAGGTTCCTGAGC |
| NCU07192_5'_fusion_2_r | ATCCACTTAACGTTACTGAAATCTCCAACGAGAACAATGAGGACTCTCG |
| 07192_promoter_f_NotI | GCGGCCGCGGATTCCTACCACACTCACTACCG |
| 07192_promoter_r_XbaI | TCTAGAGATAAGGTATGTTGTAATAAAACAG |
| pMF-Not1-Pdoc1CG3-f | cgaattggagctccaccgcggtggcggccgcGTGGAAGTGCCTGTCAC |
| Pdoc2CG3-myc-doc1CG3 | tcagaggtcctcctcggagatgagcttctgctcCGACGTCATGAACCC |
| HA-doc2CG3-r | tcaggcgtagtcggggacgtcgtaggggtaGCTATCCGCCTCTAGC |
| DOC1 CG3-myc-IGR-f | gctcatctccgaggaggacctctgaCTGTGCTGCATCCGG |
| pMF-HA-r | ggtatcgataagcttgatatcgaatTCAGGCGTAGTCGGG |
| hph F | CGGAGACAGAAGATGATATTGAAGGAGC |
| hph R | GTTGGAGATTTCAGTAACGTTAAGTGGAT |
| HygRupflank | GAATAGAGTAGATGCCGACC |
| hphp | TGCAATAGGTCAGGCTCT |
